# Low‐Dose Chemotherapy Preferentially Shapes the Ileal Microbiome and Augments the Response to Immune Checkpoint Blockade by Activating AIM2 Inflammasome in Ileal Epithelial Cells

**DOI:** 10.1002/advs.202304781

**Published:** 2024-01-08

**Authors:** Congying Pu, Yize Li, Yixian Fu, Yiyang Yan, Siyao Tao, Shuai Tang, Xiameng Gai, Ziyi Ding, Zhenjie Gan, Yingluo Liu, Siyuwei Cao, Ting Wang, Jian Ding, Jun Xu, Meiyu Geng, Min Huang

**Affiliations:** ^1^ State Key Laboratory of Drug Research Shanghai Institute of Materia Medica Chinese Academy of Sciences Shanghai 201203 China; ^2^ University of Chinese Academy of Sciences Beijing 100049 China; ^3^ School of Pharmacy, Jiangxi Medical College Nanchang University Nanchang 330031 China; ^4^ Shandong Laboratory of Yantai Drug Discovery Bohai Rim Advanced Research Institute for Drug Discovery Yantai 264117 China

**Keywords:** AIM2 inflammasome, chemotherapy, gut microbiome, ileal epithelial cells, immune checkpoint blockade, lactobacillus, segmented filamentous bacterium

## Abstract

Intervention of the gut microbiome is a promising adjuvant strategy in cancer immunotherapy. Chemotherapeutic agents are recognized for their substantial impacts on the gut microbiome, yet their therapeutic potential as microbiome modulators remains uncertain, due to the complexity of microbiome‐host‐drug interactions. Here, it is showed that low‐dose chemotherapy preferentially shapes the ileal microbiome to augment the extraintestinal immune response to anti‐programmed death‐1 (anti‐PD‐1) therapy without causing intestinal toxicity. Mechanistically, low‐dose chemotherapy causes DNA damage restricted to highly‐proliferative ileal epithelial cells, resulting in the accumulation of cytosolic dsDNA and the activation of the absent in melanoma 2 (AIM2) inflammasome. AIM2‐dependent IL‐18 secretion triggers the interplay between proximal Th1 cells and Paneth cells in ileal crypts, impairing the local antimicrobial host defense and resulting in ileal microbiome change. Intestinal epithelium‐specific knockout of AIM2 in mice significantly attenuates CPT‐11‐caused IL‐18 secretion, Paneth cell dysfunction, and ileal microbiome alteration. Moreover, AIM2 deficiency in mice or antibiotic microbial depletion attenuates chemotherapy‐augmented antitumor responses to anti‐PD1 therapy. Collectively, these findings provide mechanistic insights into how chemotherapy‐induced genomic stress is transduced to gut microbiome change and support the rationale of applying low‐dose chemotherapy as a promising adjuvant strategy in cancer immunotherapy with minimal toxicity.

## Graphical abstract

Low‐dose chemotherapy causes DNA damage restricted to highly‐proliferative ileal epithelial cells without causing intestinal toxicity, resulting in the activation of the absent in melanoma 2 (AIM2) inflammasome. AIM2‐dependent IL‐18 secretion triggers the interplay between proximal Th1 cells and Paneth cells in ileal crypts, shaping the ileal microbiome pattern and augmenting antitumor responses to anti‐PD1 therapy.

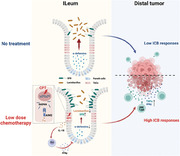

## 1 Introduction

Immune checkpoint blockade (ICB), in particular with antibodies against programmed cell death protein 1 (PD‐1), has been approved for the treatment of various malignancies, yet up to 50% of patients remain unresponsive to ICB.^[^ ^1^ ^]^ Recently, both clinical and preclinical evidence has demonstrated the association between different species of intestinal bacteria and the efficacy of ICB,^[^ ^2^, ^3^ ^]^ arousing tremendous interest in modulating the gut microbiome as a potential adjuvant for immunotherapy. Various strategies, including probiotics, prebiotics, postbiotics, antibiotics, and fecal microbiota transplantation (FMT), are being actively employed to modulate the microbiota composition for the therapeutic potential.^[^ ^4^ ^]^ However, most of these approaches, due to their broad impacts, are expected to be accompanied by risks and controversies that can potentially introduce clinical complications.^[^ ^5^, ^6^ ^]^ Recently, it has been increasingly recognized that localized microbiota alterations in the small intestine, particularly in the ileum, exert essential effects on cancer pathogenesis and therapeutic responses,^[^ ^7^, ^8^, ^9^, ^10^ ^]^ although both microbial richness and diversity in the small intestine are significantly lower than those in the large intestine. Spatial modulation of the indigenous microbes in the small intestine may inform the next‐generation strategies for microbiome‐based cancer immunotherapy.

Chemotherapy remains the mainstay of therapeutic options for patients with advanced and metastatic malignancies. Chemotherapeutic agents kill fast‐growing tumor cells via a variety of mechanisms, with most agents inducing genomic stress in tumor cells.^[^ ^11^ ^]^ Apart from killing tumor cells, chemotherapeutic agents impose a complex impact on the gastrointestinal tract, including inducing apoptosis of enterocytes, modulating intestinal immunity and remodeling the gut microbiota,^[^ ^8^, ^12^, ^13^ ^]^ suggesting that chemotherapy could be utilized as an applicable approach for gut microbial intervention. However, discrepancies exist in chemotherapy‐rendered microbial alteration and the associated patient outcomes, largely hampering its use as a microbial‐modulation tool. For example, some studies have identified a global dysbiosis following chemotherapy, leading to severe gastrointestinal and systemic toxicity,^[^ ^14^, ^15^, ^16^ ^]^ whereas others have revealed that intestine‐localized microbial composition changes caused by chemotherapy contribute to therapeutic benefit via boosting antitumor immunity.^[^ ^7^, ^17^ ^]^ Therefore, it is essential to tip the balance from chemotherapy‐caused gut toxicity toward microbiome‐rendered therapeutic benefits. To achieve this goal, it is important to reveal the molecular links between chemotherapy‐induced genomic stress and gut bacteria changes, which remain largely elusive.

This study was inspired by the prominent proliferative state of intestinal epithelial cells along the whole gut, which we believe may represent a specific vulnerability to chemotherapy. We discovered that a low‐dose chemotherapy, rather than the maximum tolerated dose (MTD) classically recommended in clinical use, can cause a localized change in the ileal microbiome that is sufficient to augment distal antitumor immunity and meanwhile avoid the potential intestinal toxicity. Herein, we provide the detailed mechanisms explaining how chemotherapy‐induced DNA damage in ileal epithelial cells is transduced to the local microbiome changes. Our findings provide new mechanistic insights into the microbiome‐host‐drug interactions and suggest low‐dose chemotherapy as a clinically applicable approach to specifically intervene in the ileal microbiome to revive antitumor immunity without causing the intestinal toxicity.

## 2 Results

### 2.1 Low‐Dose Chemotherapy Preferentially Causes DNA Damage in Ileal Epithelial Cells and Shapes the Ileal Microbiota without Causing Intestinal Toxicity

**Figure 1:**
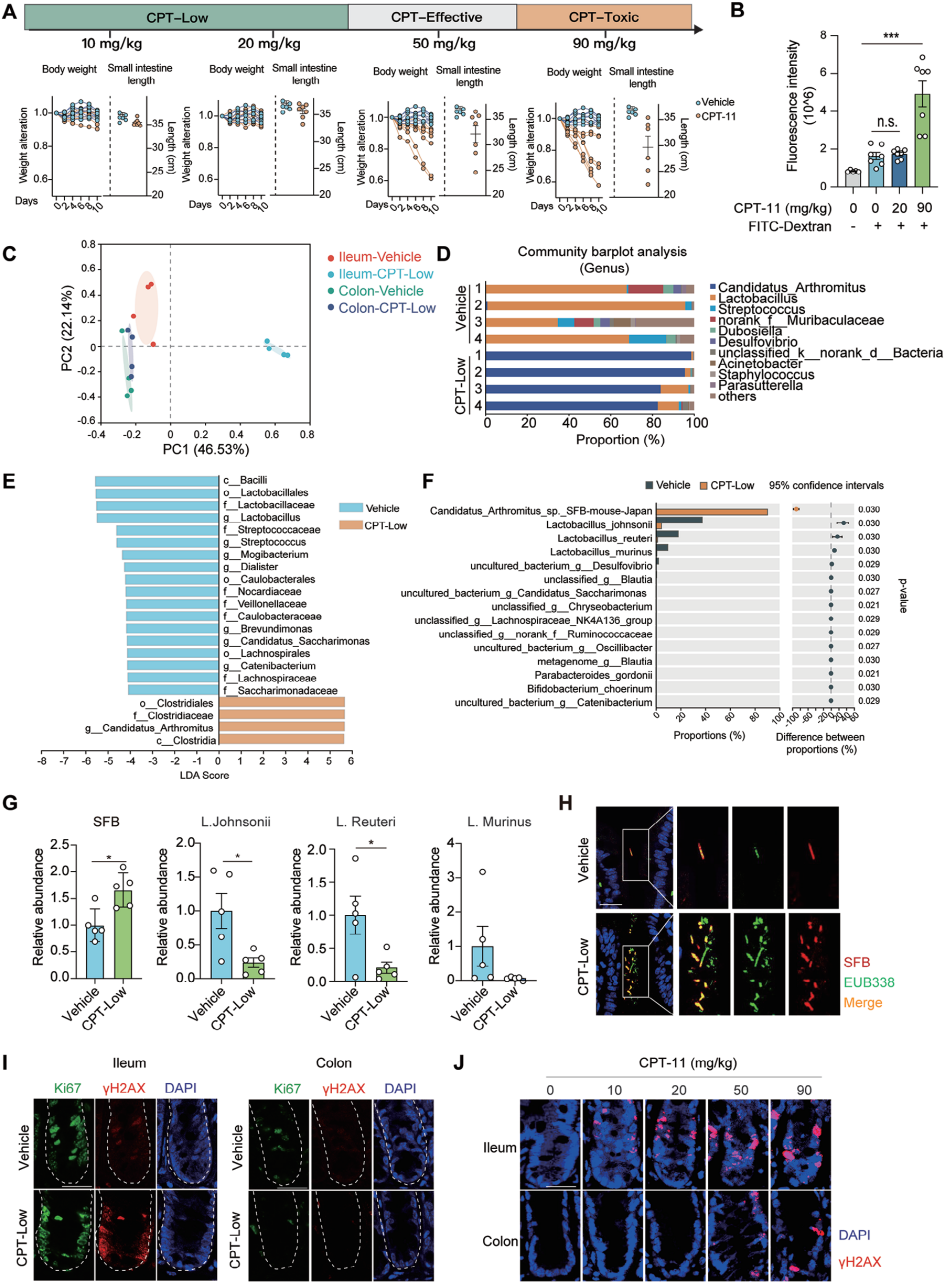
Low‐dose CPT‐11 preferentially causes DNA damage in ileal epithelial cells and shapes ileal microbiota without causing the intestinal toxicity. A) Dose‐response of CPT‐11‐caused intestinal toxicity. C57BL/6 mice were treated with different doses of CPT‐11 or vehicle control as indicated for 10 consecutive days (*n* = 6 or 7 per group). Upper, study design for dose range. Lower, mice body weight alteration and intestine length. B) Fluorescence intensity of FITC‐dextran in blood. C57BL/6 mice were treated with CPT‐11 (20 or 90 mg kg^−1^) or vehicle control for 7 consecutive days. After the last treatment, mice were fasted for 4–6 h and then treated FITC‐dextran orally at a concentration of 80 mg mL^−1^ (150 µl per mouse). C‐F) The impact of CPT‐11 on the intestinal microbiome. C57BL/6 mice were treated with CPT‐11 (20 mg kg^−1^) or vehicle control for 7 consecutive days (*n* = 4 per group). Paired colon and ileum tissues were collected for 16S rRNA gene sequencing. C) Principal coordinate analysis (PCoA) of 16S rRNA sequencing data. D) Relative proportion of ileal microbiota at the genus level. E) Differential bacterial taxonomic abundance of ileal microbiome between vehicle and CPT‐treated mice analyzed by LEfSe (linear discriminant analysis size effect) and projected as histograms. F) Genus level comparison of ileal microbiota between vehicle and CPT‐treated mice. G) Relative abundance of *segmented filamentous bacteria* (*SFB*) and *Lactobacillus* species in the ileum. C57BL/6 mice were treated as in C) (*n* = 5 per group). Genome of ileal microbiota was extracted and subjected to qPCR analysis. H) Representative images of FISH analysis of bacteria colonized in the ileal interfold regions. *SFB* was detected using a probe specifically recognizing *SFB* and total bacteria were detected using probe EUB338. Scale bar, 20 µm. I,J) Representative images of immunohistofluorescent staining of Ki67 and γH2AX. C57BL/6 mice were treated with CPT‐11 at indicated doses (20 mg kg^−1^ for I) or vehicle control for 6 h (*n* = 5 per group) and paired colon and ileum tissues were collected. Scale bar, 20 µm. Data were represented as mean ± SEM. p values were calculated by two‐tailed Student's *t*‐test. ^*^ *p* < 0.05; ^***^ *p* < 0.001.

Chemotherapeutic agents are known to severely affect the intestines. To carefully characterize the impact of chemotherapy on the intestinal microbiome, we used irinotecan (CPT‐11), a clinically widely used chemotherapeutic agent known for severely impacting the intestines in patients.^[^ ^18^, ^19^ ^]^ C57BL/6 mice were exposed to gradient doses of CPT‐11 for 10 consecutive days. Doses at approximately one twentieth (10 mg kg^−1^) to one‐tenth (20 mg kg^−1^) of MTD (≈200 mg kg^−1^ in mice for CPT‐11)^[^ ^20^ ^]^ were chosen as low doses according to the clinical definition;^[^ ^21^ ^]^ well‐documented effective dose (50 mg kg^−1^)^[^ ^22^ ^]^ and toxicity dose (90 mg kg^−1^)^[^ ^12^ ^]^ in preclinical models were included as well (**Figure** **1**A). Following the treatment, mice body weight, intestine length， morphological changes in the intestinal epithelium and microbiome alterations were examined in parallel. Consistent with clinical observations, CPT‐11 treatment tended to cause intestinal toxicity at both the effective and toxic doses, as indicated by a continuous body weight loss, the apparent shortening of the small intestine, and enormous morphological changes in the intestinal epithelium. In contrast, these effects did not occur at low doses (10 and 20 mg kg^−1^) (Figure 1A; Figure S1A,B, Supporting information). To further confirm this finding, the intestinal integrity was compared at low‐ and toxic‐dose by staining the tight junction protein ZO‐1 (Figure S1C, Supporting Information), and the intestinal permeability was measured using 4‐kDa fluorescein isothiocyanate‐dextran (FITC‐dextran) (Figure 1B). Both assays excluded the detectable intestinal toxicity following low‐dose CPT‐11 (CPT‐Low) treatment.

Of great interest, although the intestinal integrity was barely affected by CPT‐Low, the gut microbiome was effectively reshaped. 16S rRNA gene sequencing of paired ileum and colon tissues followed by principal coordinate analysis (PCoA) revealed a significantly altered overall microbial composition in the ileum upon 20 mg kg^−1^ CPT‐11 treatment, whereas negligible microbial alteration was observed in colon samples (Figure 1C). To better understand CPT‐induced ileal microbial composition alterations, the relative abundances of operational taxonomic units (OTUs) were evaluated. At the order level, *Clostridiales* was enriched in the CPT‐treated group, whereas *Lactobacillales* was enriched in the vehicle group (Figure S1D, Supporting information); at the genus level, *Lactobacillus* was enriched in the vehicle group, and *Candidatus Arthromitus*, commonly known as *segmented filamentous bacteria* (*SFB*), was significantly enriched upon CPT‐Low treatment (Figure 1D; Figure S1E, Supporting information). Linear discriminant analysis effect size (LEfSe) analysis yielded a similar result (Figure 1E; Figure S1F, Supporting Information). Further examination of the ileal microbiome at the species level identified a significant increase in *Candidatus Arthromitus sp. SFB‐mouse‐Japan*, accompanied with a consistent decrease in the abundance of *Lactobacillus* species including *Lactobacillus johnsonii*, *Lactobacillus reuteri* and *Lactobacillus murinus*, upon CPT‐Low treatment (Figure 1F). To further confirm the local microbiome change, *SFB* and *Lactobacillus* species were detected using qPCR analysis of ileum samples collected from CPT‐treated mice (Figure 1G), which identified a significantly higher amount of *SFB* and lower *Lactobacillus* species abundance in the ileum after the treatment. We also used a previously validated *SFB*‐specific probe^[^ ^23^ ^]^ to visualize the local bacterial changes in the ileum using the fluorescence in situ hybridization (FISH) analysis. Consistent with the previous findings, relatively few bacteria were located in the interfold region. Among the sparsely colonized bacteria in this region, *SFB* was relatively enriched, in agreement with the previous notion revealing their preferential colonization near the ileal mucosa.^[^ ^24^, ^25^ ^]^ Upon CPT‐Low treatment, the abundance of *SFB* near the ileal mucosa was significantly increased, constituting the major composition of the local microbiome (Figure 1H). These results indicate that low‐dose CPT‐11 specifically remodels the microbiota in the ileum, which is characterized by the increased *SFB* colonization and decreased *Lactobacillus* colonization.

We next investigated why the ileal microbiome was more susceptible to CPT‐11 treatment than the colonic microbiome. The gut epithelium is sustained by the proliferative transit amplifying cells (TACs)^[^ ^26^ ^]^ differentiated from intestinal stem cells (ISCs) lying at the base of crypts (Figure S1G left, Supporting Information). According to a previous study, cells located at the crypt base in the ileum proliferate much faster than those in the colon.^[^ ^8^ ^]^ Considering that CPT‐11 imposes genomic stress and kills tumor cells in a replication‐dependent manner, we speculated that the highly proliferative ileal TACs are the major target cells of CPT‐Low and may trigger local microbe alterations. To test this possibility, the proliferative state indicated by Ki67 staining, as well as the drug‐caused DNA double‐strand breaks (DSBs) indicated by γ‐H2AX staining, were concurrently examined in intestinal tissues from C57BL/6 mice. In the region where ileal TACs are located, cells showed strong signaling of Ki67 staining, much higher than that in the crypt of colon (Figure 1I). Consistent with the proliferative state, drug‐induced DNA double‐strand breaks (DSBs) specifically occurred in Ki67‐positive cells in the ileum (Figure 1I). These data suggest that ileal proliferative cells in the intestines are more susceptible to chemotherapy. To confirm this result, γ‐H2AX staining was carried out in Lgr5‐EGFP mice, in which Lgr5‐positive ISCs were visualized by EGFP fluorescence. CPT‐induced DNA damage in ileal crypts was restricted to cells residing above the Lgr5‐positive ISCs, consistent with the location of TACs (Figure S1G, Supporting Information). Furthermore, we compared the impact of different dosages of CPT‐11. At lower doses, CPT‐11 exclusively induced an ileum‐restricted DNA damage, whereas treatment at higher doses caused massive DNA damage in both ileum and colon tissues (Figure 1J). These data demonstrate that low‐dose CPT‐11 specifically induces genomic stress in proliferative ileal epithelial cells, which is associated with ileum‐specific microbiome modulation.

### 2.2 Ileum‐Specific Microbiome Alteration Activates Extraintestinal Antitumor Immunity and Augments the Response to Anti‐PD1 Therapy

**Figure 2:**
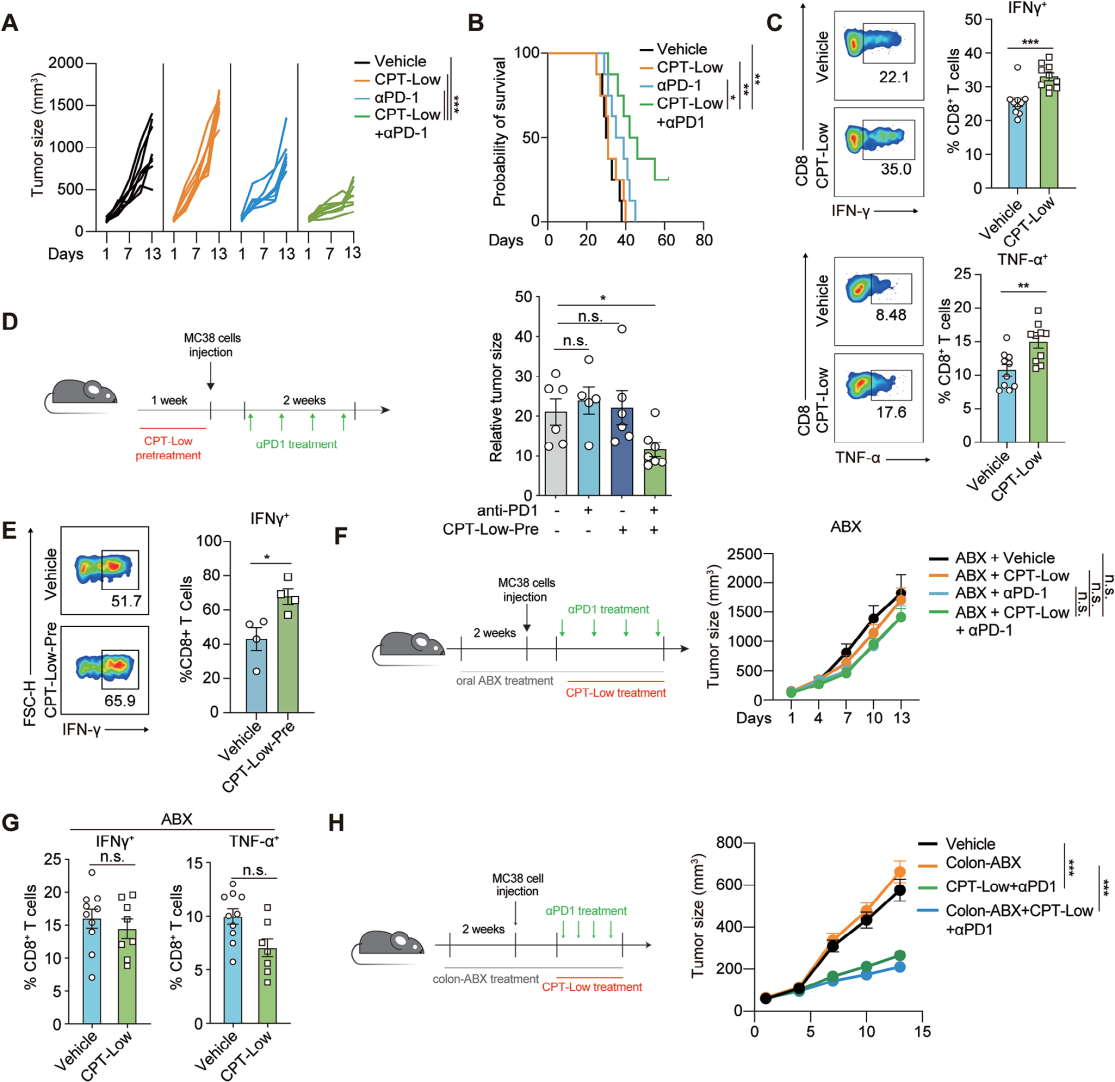
Ileum‐specific microbiome alteration activates extraintestinal antitumor immunity and augments the response to anti‐PD1 therapy. A,B) Combinational effect of CPT‐Low and anti‐PD‐1 therapy. MC38 tumor‐bearing C57BL/6 mice were treated with anti‐PD‐1 antibody (5 mg kg^−1^, twice per week) alone or in combination with CPT‐Low (20 mg kg^−1^, daily) for indicated days (*n* = 8 per group). A) Tumor growth curve of individual mouse. B) Mice survival analysis. C) The proportion of IFN‐γ or TNF‐α positive cells in CD8^+^ T cells in tumor infiltrated lymphocytes. MC38 tumor‐bearing C57BL/6 mice were treated with CPT‐Low (20 mg kg^−1^) or vehicle control for 7 consecutive days (*n* = 10 per group). D,E) The impact of pre‐treatment of CPT‐Low on anti‐PD1 therapy. C57BL/6 mice were treated with CPT‐Low or vehicle control for one week followed by MC38 tumor inoculation. Anti‐PD‐1 antibody (5 mg kg^−1^) was given twice per week when tumor volume reaching 50–80 mm^3^. D) Left, scheme showing the treatment procedure. Right, relative tumor size at the endpoint (*n* = 6 to 8 per group). E) The proportion of IFN‐γ positive cells in CD8^+^ T cells in tumor infiltrated lymphocytes (*n* = 4 per group). F,G) The impact of microbiome depletion via orally‐given ABX on the combinational effect of CPT‐Low and anti‐PD‐1 therapy. F) Left, scheme showing the treatment procedure. Right, tumor growth curve (*n* = 5 or 6 per group). G) The proportion of IFN‐γ positive cells in CD8^+^ T cells in tumor infiltrated lymphocytes (*n* = 8 or 10 per group). H) The impact of colon‐specific microbiome depletion on the combinational effect of CPT‐Low and anti‐PD‐1 therapy. Left, scheme showing the treatment procedure. Right, tumor growth curve (*n* = 9 or 10 per group). Data were represented as mean ± SEM. p values were calculated by two‐sided Student's *t*‐test or by log‐rank test. n.s., not significant; ^*^ *p* < 0.05; ^**^ *p* < 0.01; ^***^ *p* < 0.001.

Both *SFB* and *Lactobacillus* are known to preferentially colonize in the small intestine, especially in the terminal ileum.^[^ ^27^ ^]^ Of note, the enrichment of *SFB* is known to exhibit a proinflammatory capability,^[^ ^25^, ^28^ ^]^ while *Lactobacillus* tends to shape a systemic anti‐inflammatory state,^[^ ^29^, ^30^ ^]^ both of which are closely associated with the host immunity. We hence asked whether CPT‐induced ileum‐specific microbiome changes, characterized by concurrent *SFB* enrichment and *Lactobacillus* depletion could favor the remote antitumor immune response to anti‐PD1 therapy. To this end, the combination of CPT‐Low or suboptimal dose of anti‐PD‐1 antibody (5 mg kg^−1^) was tested in colon adenocarcinoma MC38 mouse syngeneic tumor models. Although monotherapy with either CPT‐Low or anti‐PD‐1 antibody barely achieved the apparent therapeutic effect, their combination significantly suppressed the tumor growth (**Figure** **2**A) and prolonged the survival of tumor‐bearing mice (Figure 2B). Analysis of the tumor‐infiltrating lymphocytes (TILs) in CPT‐treated tumors showed the augmented function of CD8^+^ T cells, as indicated by the increased expression of interferon‐γ (IFN‐γ) and TNF‐α in CD8^+^ T cells (Figure 2C). Moreover, the synergistic effect of CPT‐Low with anti‐PD1 antibody was abolished in nude mice, confirming the involvement of the augmented adaptive immunity (Figure S2A, Supporting information).

To investigate whether chemotherapy‐shaped host immune status, rather than a direct impact of chemotherapy on the tumor microenvironment, accounted for the enhanced response to anti‐PD‐1 therapy, we carried out a sequential treatment experiment. To this end, MC38 tumor‐bearing mice were treated with low‐dose CPT‐11 for one week and then switched to anti‐PD‐1 antibody in the following week, or the two treatments were administered in the reverse order (Figure S2B, Supporting Information). Notably, only pretreatment with CPT‐Low was able to synergize with PD‐1 blockade in controlling tumor growth (Figure S2C, Supporting Information). Furthermore, CPT‐Low was administered even prior to the tumor implantation, which similarly sensitized MC38 tumors to anti‐PD1 antibody (Figure 2D) and augmented intratumoral CD8^+^ T‐cell function (Figure 2E). These results suggest that chemotherapy‐modulated host immune status is sufficient to enhance the efficacy of anti‐PD‐1 therapy.

We further demonstrated the contribution of the CPT‐reshaped‐ileal microbiome to the therapeutic benefit of PD‐1 blockade. As *SFB* culture and the generation of *SFB*‐monocolonized mice is extremely technically challenging,^[^ ^31^ ^]^ and as mouse lines supporting microbiome transplantation of *SFB* ^[^ ^25^ ^]^ are not available in China, we took an alternative approach of antibiotic treatment. Systemic and regional depletion of gut microbes using antibiotics were conducted in parallel. To this end, tumor‐bearing mice were orally pretreated with an antibiotic mixture (ABX) to systemically disrupt the gut microbiome (Figure S2D, Supporting Information). The synergy between CPT‐Low and anti‐PD1 antibody was completely abolished by the pretreatment with ABX (Figure 2F). Likewise, the CPT‐enhanced function of tumor‐infiltrated CD8^+^ T cells was largely attenuated as well (Figure 2G). To further strengthen the contribution of the ileal microbiome, the colon microbiome was specifically depleted using colonic gavage with ABX. Importantly, we discovered that the depletion of the colon microbiome (Figure S2E, Supporting Information) failed to attenuate the therapeutic effect of the CPT‐Low and anti‐PD‐1 combination (Figure 2H). These data together suggest that low‐dose CPT‐11 stimulates antitumor immunity and synergizes with anti‐PD‐1 therapy via causing ileum‐specific microbiome alterations.

### 2.3 Ileal Epithelium‐Restricted Activation of AIM2 Inflammasome Shapes the Indigenous Microbiome

**Figure 3:**
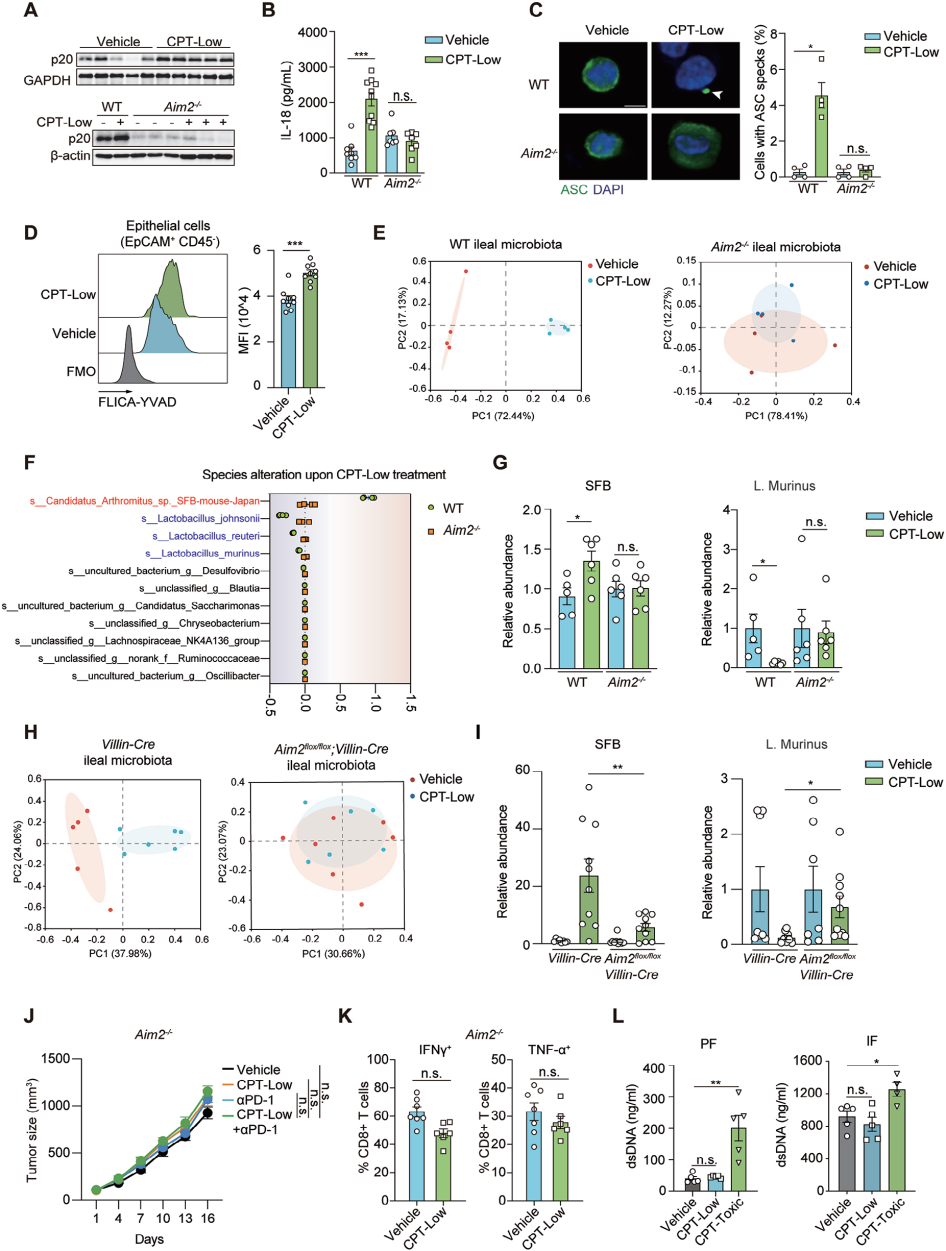
Ileal epithelium‐restricted activation of AIM2 inflammasome shapes the indigenous microbiome. A–C) AIM2 inflammasome activation in the ileum. *Aim2* ^−/−^ and littermate wildtype mice (WT) were treated with CPT‐Low (20 mg kg^−1^) or vehicle control for 7 consecutive days. A) Immunoblotting analysis of cleaved caspase‐1 (p20) in the ileum lysate (*n* = 3 or 5 per group). B) ELISA analysis of IL‐18 in the ileum lysate (*n* = 7 to 10 per group). C) Immunofluorescent staining of ASC specks in ileal crypt cells. Cells were isolated from the ileal crypts collected from mice receiving the indicated treatment (*n* = 4 per group). Quantification of cells with ASC specks was normalized by total cell counts. Arrows indicate ASC specks. Scale bar, 5 µm. D) Caspase‐1 activity assessed by a FLICA (fluorescent labelled inhibitor of caspase‐1) probe. C57BL/6 mice were treated with CPT‐Low (20 mg kg^−1^) or vehicle control for 7 consecutive days (*n* = 8 per group) and EpCAM^+^CD45^−^ epithelial cells digested from ileal crypts were subjected to FLICA staining. Show are representative flow cytometry histogram (left) and relative mean fluorescence intensity (MFI). Fluorescence minus one (FMO) control was used to define negative staining for FLICA probe. E–G) The impact of AIM2 deficiency on ileal microbiome alteration. *Aim2* ^−/‐^ and littermate WT mice were treated as in A). Ileum tissues were collected for 16S rRNA gene sequencing (*n* = 4 per group) or bacterial qPCR analysis (*n* = 5 or 6 per group). E) Principal coordinate analysis (PCoA). F) Abundance alteration of top‐10 abundant bacteria at the species level. G) Relative abundance of *SFB* and *Lactobacillus* species measured by qPCR analysis. H,I) The impact of AIM2 deficiency in the intestinal epithelial cells on microbiome alteration. Intestinal epithelial cell‐specific AIM2 knockout mice (*Aim2 ^flox/flox^;Villin‐Cre*) or littermate control (*Villin‐Cre*) were treated as in A) (*n* = 7 to 10 per group) and ileum tissues were collected for 16S rRNA sequencing analysis (H) or microbial qPCR analysis (I). H) Principal coordinate analysis (PCoA) (*n* = 5 or 6 per group). I) Relative abundance of *SFB* and *Lactobacillus* species measured by qPCR. J) Tumor growth curve showing the impact of AIM2 deficiency on the combinational effect of CPT‐Low and anti‐PD1 therapy. *Aim2* ^−/−^ mice bearing MC38 tumors were treated with anti‐PD‐1 antibody (5 mg kg^−1^, twice per week) alone or in combination with CPT‐Low (20 mg kg^−1^, daily) (*n* = 6 or 7 per group). K) IFN‐γ^+^ or TNF‐α^+^ cell ratio in CD8^+^ T cells in tumor infiltrated lymphocytes. *Aim2* ^−/−^ mice bearing MC38 tumors were treated with CPT‐Low (20 mg kg^−1^) or vehicle control for 7 consecutive days (*n* = 6 or 7 per group). L) dsDNA concentration in cell‐depleted peritoneal lavage fluid (PF) and ileum flushed fluid (IF). C57BL/6 mice were treated as in A) (*n* = 4 or 5 per group). Data were represented as mean ± SEM. p values were calculated by two‐tailed Student's *t*‐test. n.s., not significant; ^*^ *p* < 0.05; ^**^ *p* < 0.01; ^***^ *p* < 0.001.

We next investigated how chemotherapy‐induced ileum‐specific DNA damage was transmitted to shape the indigenous microbiome. Absent in melanoma 2 (AIM2) inflammasome, a multiprotein platform that recognizes aberrant cytosolic double‐stranded DNA (dsDNA) to induce proinflammatory cytokine production and pyroptosis,^[^ ^32^ ^]^ is known to play an essential role in governing the intestinal immunity.^[^ ^33^ ^]^ Recently, accumulating evidence has revealed the causal link between chemotherapy‐induced DNA damage and AIM2 activation.^[^ ^12^, ^34^, ^35^ ^]^ We hence investigated whether AIM2 inflammasome activation is involved in CPT‐11‐induced ileal microbiome alterations. Activation of ileal AIM2 inflammasome was assessed by measuring the cleavage of caspase‐1 and the production of interleukin‐18 (IL‐18). In ileum tissues collected from CPT‐treated mice, drug treatment clearly increased the caspase‐1 cleavage (**Figure** **3**A) and the secretion of IL‐18 (Figure 3B). These changes were all abolished in *Aim2*‐deficient mice (Figure 3A,B). Furthermore, ileal crypt cells were isolated from *Aim2*‐knockout and wild‐type (WT) mice similarly receiving CPT‐Low treatment. We discovered that ASC specks, a specific indicator of the inflammasome assembly, were detected in cells derived from CPT‐treated WT mice but not in those from *Aim2*‐deficient mice (Figure 3C). To confirm intestinal epithelial‐specific AIM2 activation, caspase‐1 activity was measured in total single cells digested from ileal crypts using a FLICA (fluorescent labelled inhibitor of caspase‐1) probe.^[^ ^36^ ^]^ It was observed that caspase‐1 activity in intestinal epithelial cells (EpCAM^+^CD45^−^) was significantly enhanced upon low‐dose CPT‐11 treatment (Figure 3D). In contrast, EpCAM^−^CD45^+^ cells indicative of intestinal intraepithelial lymphocytes were barely affected (Figure S3A, Supporting Information). Considering that lamina propria (LP) immune cells were shown as a major source of AIM2 inflammasome activation upon treatment of toxic‐dose CPT‐11,^[^ ^12^ ^]^ digested LP single cells were obtained and the FLICA signal was specifically analyzed for LP‐resident macrophage, cDC1 and cDC2, in which AIM2 inflammasome is highly expressed. We discovered that the impact of low‐dose CPT‐11 on AIM2 inflammasome was very marginal (Figure S3B, Supporting Information). These results suggest that AIM2 activation induced by low‐dose CPT‐11 is mostly ileal epithelium‐restricted.

AIM2 is activated by cytosolic dsDNA. As chemotherapy‐induced genomic stress could cause the aberrant accumulation of dsDNA in the cytosol,^[^ ^37^ ^]^ we asked whether it was the reason for ileal AIM2 activation. To this end, ileal crypt cells were isolated from CPT‐11‐ or vehicle‐treated mice and then stained for dsDNA using a well‐documented anti‐dsDNA antibody.^[^ ^38^, ^39^ ^]^ Upon CPT‐low treatment, cytosolic dsDNA signaling was present in the extranuclear regions (Figure S3C, Supporting information), consistent with previously reported cytosolic dsDNA staining pattern.^[^ ^38^, ^39^ ^]^ To further establish the causal link between chemotherapy‐induced cytosolic dsDNA production and AIM2 activation, we performed a set of in vitro assays using the human embryonic kidney (HEK) 293T model cell line, which lacks the endogenous AIM2 inflammasome machinery. In these cells, the treatment with SN38, an in vivo active metabolite of CPT‐11, caused the accumulation of cytosolic dsDNA, which was rescued when DNA replication was stalled by serum starvation, as CPT‐11 induces DNA damage in a replication‐dependent manner (Figure S3D, Supporting Information). Importantly, in HEK 293T cells reconstituted with AIM2, SN38‐activated ASC speck formation (Figure S3E, Supporting Information) was similarly abolished by serum starvation, further supporting that CPT‐provoked cytosolic dsDNA production leads to AIM2 inflammasome activation. Similar results were obtained by the treatment with cisplatin, a DNA‐damaging agent, which similarly took effect in a replication‐dependent manner (Figure S3F, Supporting Information). These results suggest that chemotherapy‐induced genomic stress in replicative ileal epithelial cells results in the cytosolic accumulation of dsDNA to activate AIM2 signaling.

To examine whether AIM2 inflammasome activation contributed to the observed microbiota alteration, 16S rRNA sequencing was performed to compare the ileal microbiome alteration in *Aim2‐defi*cient mice and their WT littermates. Notably, CPT‐rendered alteration of the ileal microbiome in WT mice was completely abolished in *Aim2‐defi*cient littermates (Figure 3E). Further assessment of the microbial landscape revealed that, in great contrast to the case in WT mice upon CPT‐Low treatment, AIM2 deficiency in mice largely reversed the increased colonization of *SFB* and the decreased colonization of *Lactobacillus* in the ileum (Figure 3F; Figure S3G, Supporting Information). Similar results were obtained using bacterial qPCR analysis (Figure 3G). To confirm the causal link between intestinal epithelial AIM2 activation and the observed ileal microbiome change, we examined the impact of CPT‐Low on intestinal epithelium‐specific AIM2 knockout mice (*Aim2 ^flox/flox^;Villin‐Cre*). In agreement with the abolished caspase‐1 activation in intestinal epithelial cells (Figure S3H, Supporting Information), ileal microbiome alteration induced by low‐dose CPT‐11 was largely abrogated in these mice (Figure 3H,I; Figure S3I–K, Supporting Information). Consistent with the attenuated microbiome change, in *Aim2*‐deficient mice, CPT‐Low failed to sensitize MC38 tumors to anti‐PD1 therapy (Figure 3J) or enhance the functions of intratumoral CD8^+^ T cells (Figure 3K). These data demonstrate that AIM2 inflammasome activation is required for the CPT‐shaped ileal microbiome and the associated antitumor immune response.

Our previous study has revealed that CPT‐11 at a high‐dose (90 mg kg^−1^) induces massive intestinal epithelial cell apoptosis, releasing large quantities of dsDNA that are taken up by surrounding immune cells to activate cytosolic AIM2, resulting in the intestinal toxicity.^[^ ^12^ ^]^ To dissect the difference between high‐ and low‐dose treatment, we performed in situ terminal deoxynucleotidyl transferase‐mediated deoxyuridine triphosphate nick end labeling (TUNEL) staining in ileum tissues. Very few cells in the ileal crypt region were stained positive after low‐dose CPT‐11 treatment, in great contrast to the massive cell epithelial cell death detected at the high‐dose^[^ ^12^, ^40^ ^]^ (Figure S3L, Supporting information). In support of this result, low‐dose CPT‐11 barely induced dsDNA release into the peritoneal lavage fluid or the fluid flushed from ileum tissues, while a significant amount of dsDNA was detected in the samples derived from the high‐dose treated mice (Figure 3L). These results suggest that whether AIM2 activation is restricted to ileal epithelial cells is the key difference between the low‐ and high‐dose chemotherapy. Low‐dose chemotherapy causes ileum‐restricted DNA damage that enables avoidance of massive intestinal toxicity.

Altogether, these data suggest that low‐dose chemotherapy results in an ileal epithelium restricted AIM2 activation, leading to ileal microbiome alterations that are able to augment the remote antitumor immunity.

### 2.4 Ileal Epithelial AIM2 Activation Diminishes the Antibacterial Function of Paneth Cells

**Figure 4:**
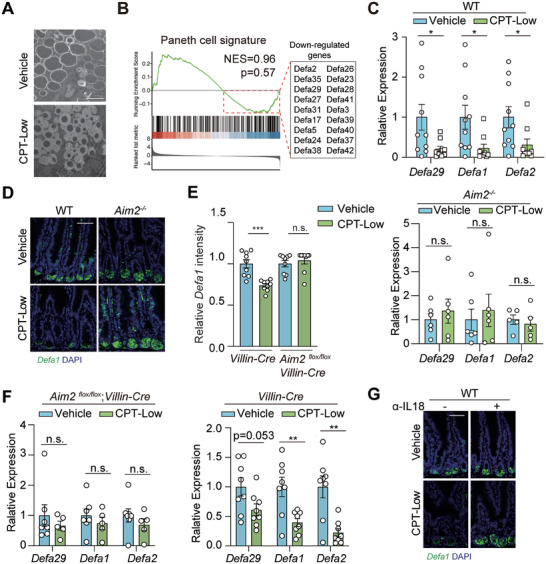
Ileal epithelial AIM2 activation diminishes the antibacterial function of Paneth cells. A,B) Paneth cell function analysis. C57BL/6 mice were treated with CPT‐Low (20 mg kg^−1^) or vehicle control for 7 consecutive days (*n* = 4 per group). A) Representative images of transmission electron microscopy analysis of ileal Paneth cells at the base of ileal crypts. Scale bar, 2 µm. B) GSEA of RNA‐seq data of the isolated ileal crypts for Paneth cell signatures. Down‐regulation of α‐defensin upon CPT‐Low treatment was marked in the box. C, D) α‐defensin production analysis in the ileum. *Aim2^−/−^* and littermate wildtype (WT) mice were treated with CPT‐Low (20 mg kg^−1^) or vehicle control for 7 consecutive days (*n* = 8 or 10 per group for WT mice; *n* = 5 or 6 per group for *Aim2* ^−/−^ mice) and ileum tissues were harvested. C) Relative mRNA expression of α‐defensins. Expression level of *Defa29, Defa1* and *Defa2* was measured by RT‐qPCR. D) Representative images of *Defa1* mRNA analyzed by RNA FISH. Scale bar, 50 µm. E,F) α‐defensin production in the intestinal epithelial cell‐specific AIM2 knockout mice. *Aim2 ^flox/flox^;Villin‐Cre* or littermate control (*Villin‐Cre*) mice were treated as in C) (*n* = 7 or 8 per group). E) Relative quantification of the intensity of *Defa1* mRNA analyzed by RNA FISH assay. F) Relative expression level of *Defa29, Defa1* and *Defa2* measured by RT‐qPCR. G) Representative images of FISH analysis of *Defa1* mRNA in the ileum. Scale bar, 50 µm. C57BL/6 mice received anti‐IL‐18 antibody (10 mg kg^−1^) were treated with CPT‐Low (20 mg kg^−1^) or vehicle control for 7 consecutive days and ileal crypts were collected for analysis. Data were represented as mean ± SEM. p values were calculated by two‐tailed Student's *t*‐test. n.s., not significant; ^*^ *p* < 0.05, ^**^ *p* < 0.01; ^***^ *p* < 0.001.

It remained unclear how AIM2 inflammasome activation in ileal epithelial cells modulates the ileal microbiome. Paneth cells, a subset of specialized secretory epithelial cells located at the bottom of small intestinal crypts, play a crucial role in shaping local microbiome via producing multiple antimicrobial peptides (AMPs).^[^ ^41^, ^42^, ^43^ ^]^ We hence examined the in situ status of Paneth cells in the ileum. In CPT‐treated mice, the lysozyme expression (Figure S4A, Supporting Information) and Alcian blue staining (Figure S4B,C, Supporting Information) of ileum tissues revealed the negligible numerical alteration of Paneth cells after chemotherapy, largely excluding the possibility of Paneth cell loss. However, upon CPT‐Low treatment, Paneth cells exhibited altered lysozyme allocation, with the majority showing diffuse lysozyme staining (Figure S4D, Supporting Information). We hence performed the transmission electron microscopy analysis of ileum tissues derived from drug‐treated mice, which revealed the abnormal Paneth cell granules, with a dramatic decrease in the numbers of electron‐dense secretory vesicles at the base of crypts (**Figure** **4**A). Further RNA‐seq analysis of isolated ileal crypts demonstrated a marginal alteration of Paneth cell signatures^[^ ^44^ ^]^ but a significant downregulation of α‐defensin,^[^ ^24^ ^]^ a type of AMPs exclusively produced by small intestinal Paneth cells (Figure 4B). Downregulation of α‐defensin by low‐dose CPT‐11 was further confirmed by RT‐qPCR analysis (Figure 4C), as well as in situ hybridization of *Defa1* mRNA (Figure 4D). These results confirmed the Paneth cell abnormality rather than cell loss upon CPT‐Low treatment.

To examine whether CPT‐induced Paneth cell dysfunction was due to AIM2 activation, we compared the α‐defensin levels between WT and *Aim2‐*knockout ileum tissues. Importantly, *Aim2* deficiency significantly attenuated the downregulation of α‐defensin caused by CPT‐Low (Figure 4C,D). These findings were recapitulated in intestinal epithelial cell‐specific AIM2 knockout mice (Figure 4E,F; Figure S4E, Supporting Information). Similarly, IL‐18 neutralization in mice reversed CPT‐induced α‐defensin downregulation (Figure 4G; Figure S4F, Supporting Information). These data suggest that the activation of the AIM2‐IL‐18 axis induces Paneth cell dysfunction, characterized by α‐defensin downregulation, which may account for the alteration of local microbiome upon low‐dose CPT‐11 treatment.

### 2.5 AIM2‐Dependent IL‐18 Secretion Triggers Ileal Th1‐Paneth Cell Interplay to Facilitate *SFB* Overgrowth and *Lactobacillus* Depletion

**Figure 5:**
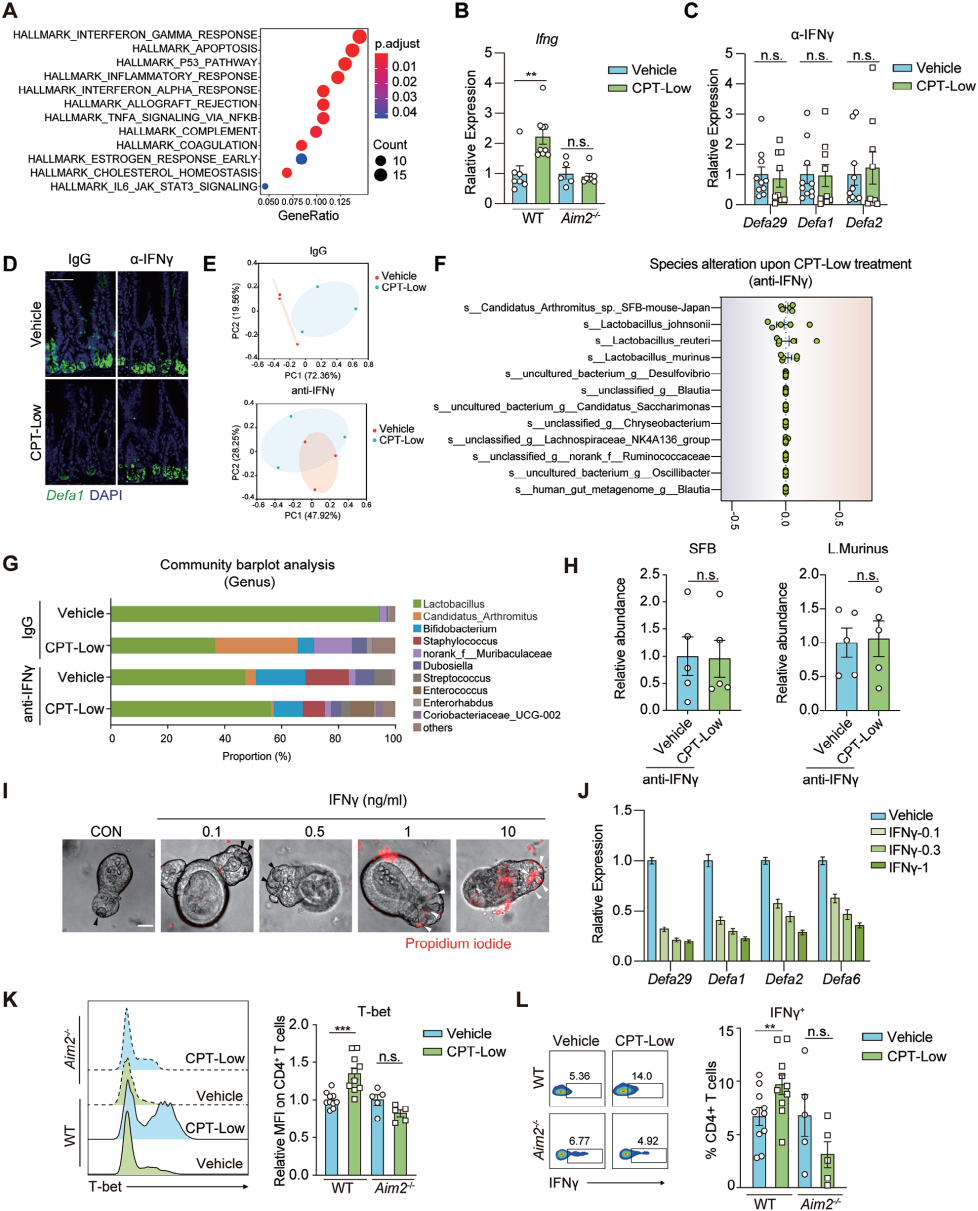
AIM2‐dependent IL‐18 secretion triggers ileal Th1‐Paneth cell interplay to facilitate *SFB* overgrowth and *Lactobacillus* depletion. A) Pathway enrichment of CPT‐upregulated genes. C57BL/6 mice were treated with CPT‐Low (20 mg kg^−1^) or vehicle control for 7 consecutive days (*n* = 4 per group) and ileal crypts were collected for RNA‐seq analysis. B) Relative mRNA expression of *Ifng* in ileum tissues. *Aim2^−/−^* and littermate wildtype (WT) mice were treated as in A) (*n* = 5 to 9 per group) and the expression level of *Ifng* was measured by RT‐qPCR analysis. C,D) The impact of IFN‐γ blockage on CPT‐impaired α‐defensin production. C57BL/6 mice received anti‐IFN‐γ antibody (10 mg kg^−1^) were treated with CPT‐Low (20 mg kg^−1^) or vehicle control for 7 consecutive days and ileal crypts were collected. C) Relative expression level of α‐defensins measured by RT‐qPCR analysis (*n* = 8 or 10 per group). D) Representative images of FISH analysis of *Defa1* mRNA (*n* = 5 per group). Scale bar, 50 µm. E–H) The impact of IFN‐γ blockage on CPT‐caused ileal microbiome alteration. C57BL/6 mice were treated as in C) (*n* = 3 or 5 per group) and ileum tissues were collected for 16S rRNA sequencing analysis (E‐G) or microbial qPCR analysis (H). E) Principal coordinate analysis (PCoA). F) Abundance alteration of top‐10 abundant bacteria at the species level. G) Relative proportion of ileal microbiota at the genus level. H) The abundance of *SFB* and *Lactobacillus.murinus* measured by qPCR. I,J) The impact of IFN‐γ on cell viability and α‐defensin production of Paneth cells. Small intestinal organoids were treated with IFN‐γ as indicated for 48 h. Shown are representative results of at least three independent experiments. I) Representative images of propidium iodide staining. J) Relative mRNA expression of α‐defensins. K,L) CPT‐caused Th1 activation in the ileum. *Aim2^−/−^* and littermate WT mice were treated as in A) and ileal lamina propria lymphocytes were isolated for flow cytometry analysis (*n* = 5 to 9 per group). K) Representative flow cytometry histogram (left) and quantification for mean fluorescence intensity (MFI) (right) of T‐bet on CD4^+^ T cells. L) Representative flow cytometry plot (left) or quantification (right) for IFN‐γ^+^ cell ratio in CD4^+^ T cells. Data were represented as mean ± SEM. p values were evaluated by two‐tailed Student's *t*‐test. n.s., not significant; ^**^ *p* < 0.01; ^***^ *p* < 0.001.

To further understand how AIM2 activation in ileal epithelial cells is mechanistically linked to the proximal Paneth cells, we performed RNA‐seq analysis of the isolated ileal crypts from CPT‐treated mice. A total of 131 genes upregulated by CPT‐Low were subjected to the pathway enrichment analysis using hallmark gene sets extracted from the molecular signatures database (MSigDB).^[^ ^45^ ^]^ Among the enriched pathways, the hallmark of IFN‐γ response was listed as the top affected pathway (**Figure** **5**A). RT‐qPCR analysis of the ileum tissues further confirmed the upregulation of *Ifng* expression level upon CPT‐Low treatment and that change was abolished in *Aim2*‐deficient mice (Figure 5B). To determine whether AIM2‐dependent of IFN‐γ production accounted for Paneth cell dysfunction, IFN‐γ was blocked using an anti‐IFN‐γ neutralizing antibody in mice (Figure 5C,D), which largely reversed the downregulation of α‐defensin in the ileum in CPT‐treated mice. Likewise, pharmacological inhibition of IFN‐γ downstream JAK1/2‐STAT signaling using JAK1/2 inhibitor INCB018424 yielded the similar results (Figure S5A, Supporting information). Along with the restoration of Paneth cell function, 16S rRNA sequencing revealed that IFN‐γ blockade significantly abolished CPT‐rendered ileal microbial remodeling (Figure 5E), as manifested by the reversal of the *SFB* enrichment and *Lactobacillus* decrease in the ileum in CPT‐treated mice (Figure 5F,G; Figure S5B, Supporting Information). Similar results were obtained using bacterial qPCR analysis (Figure 5H). These data confirmed that IFN‐γ signaling is critical for CPT‐induced ileal microbiome modulation.

To confirm whether IFN‐γ directly affected the function of Paneth cells, small intestinal organoids were established and exposed to different concentrations of IFN‐γ. Consistent with the finding of previous studies,^[^ ^46^ ^]^ a rapid cell death and Paneth cell loss were induced by IFN‐γ at concentrations ranging from 1 to 10 ng ml^−1^ (Figure 5I). However, these concentrations seemed much higher than the IFN‐γ level detected in the ileal homogenate derived from CPT‐treated mice, as detected by the enzyme‐linked immunosorbent assay (ELISA) (Figure S5C, Supporting information). We thus focused to IFN‐γ at lower concentrations (0.1–0.3 ng ml^−1^). Low‐concentration IFN‐γ stimulation decreased the expression of α‐defensin (Figure 5J) without affecting Paneth cell survival (Figure 5I), in agreement with our findings in vivo. These results suggest that ileal AIM2 activation impairs the antimicrobial functions of proximal Paneth cells via a mechanism dependent on IFN‐γ‐JAK1/2 signaling.

The molecular links between AIM2 activation and IFN‐γ signaling remain unknown. In close proximity to the crypt base, lamina propria (LP)‐resident T helper 1 (Th1) cells, a CD4^+^ T cell subset, are the major sources of intestinal IFN‐γ.^[^ ^47^ ^]^) While T helper 17 (Th17) and regulatory T cells (Tregs), two other CD4^+^ T cell subsets residing in the lamina propria, were not significantly affected (Figure S5D,E, Supporting Information), the proportion of Th1 cells was increased after CPT‐Low treatment, as indicated by the increased expression of T‐bet, the master regulator of Th1 differentiation (Figure 5K). Further analysis of Th1 cells in the ileal lamina propria revealed the increased IFN‐γ expression in CPT‐treated WT mice (Figure 5L). Moreover, the proportion of Th1 cells and IFN‐γ expression in mesenteric lymph nodes (mLNs) were increased as well (Figure S5F, Supporting Information), implying an active mobilization of Th1 cells upon chemotherapy. In contrast, in *Aim2*‐deficient mice, CPT‐boosted Th1 immunity was largely diminished (Figure 5K,L; Figure S5F, Supporting Information), suggesting that AIM2 activation leads to the stimulation of Th1 immunity in the ileum. AIM2 downstream IL‐18 has been recognized as a Th1‐inducing cytokine.^[^ ^48^ ^]^ We thus used a neutralizing antibody against IL‐18 to examine whether it was functionally involved. Upon IL‐18 blockade in vivo, CPT‐caused Th1 induction in both the ileum (Figure S5G, Supporting Information) and mLNs (Figure S5H, Supporting Information) were largely impeded. Similar results were obtained by analyzing the function of intratumoral CD8^+^ T cells (Figure S5I, Supporting Information). These results confirmed that the AIM2‐IL‐18 axis‐orchestrated ileal epithelial‐Th1‐Paneth cell interplay is responsible for chemotherapy‐induced ileal microbiome alterations and the resultant antitumor immune response.

**Figure 6:**
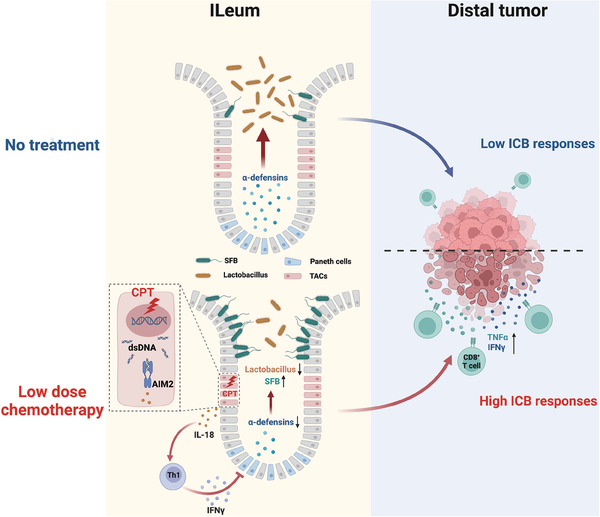
Diagram depicting low‐dose chemotherapy‐activated compartmental cell crosstalk in shaping the ileal microbiome and dictating the therapeutic benefit of immune checkpoint blockade. Low‐dose chemotherapy induces DNA damage restricted in ileal TACs, which causes cytosolic dsDNA accumulation and activates dsDNA‐sensor AIM2 inflammasome in these cells. AIM2‐dependent production of IL‐18 further triggers the interplay between proximal Th1 and Paneth cells, resulting in the impaired α‐defensin production by Paneth cells and an ileal microbial pattern characterized by *SFB* outgrowth and *Lactobacillus* depletion. The altered ileal microbiome further shapes host immunity, rendering distal tumor microenvironment more responsive to ICB therapy. TACs, transit amplifying cells; dsDNA, double‐stranded DNA; *SFB*, *segmented filamentous bacterium*; ICB, immune checkpoint blockade.

Together, our findings reveal the susceptibility of proliferative ileal epithelial cells to low‐dose chemotherapy and provide the missing links between chemotherapeutic agent‐caused genomic stress and the intestinal microbiome alteration. According to our findings, low‐dose chemotherapy preferentially induces DNA damage and the accumulation of cytosolic dsDNA in ileal epithelial cells, which activates AIM2 inflammasome signaling to trigger cell‐cell crosstalk in the small intestine. In this multicell interplay, the secretion of IL‐18 links the intracellular AIM2 inflammasome to the proximal Th1 cells residing in the ileal lamina propria, which further affects Paneth cells in the ileal crypt via secreting IFN‐γ. This interplay shapes the indigenous microbiome characterized by *SFB* overgrowth and *Lactobacillus* depletion (**Figure** **6**). Our findings highlight low‐dose chemotherapy as a spatial microbial modulator and an adjuvant strategy for cancer immunotherapy.

## 3 Discussion

The intimate association between gut microbial composition and the efficacy of ICB therapy has motivated a great enthusiasm in intervening the gut microbiota for therapeutic potential, yet the current strategies lack a spatial specificity, adding a large uncertainty to the therapeutic outcome. In this study, we identified low‐dose chemotherapy as a ready‐to‐use intervention approach to specifically intervene in the ileal microbiome to enhance ICB efficacy. Low‐dose treatment allows a relatively localized and moderate change of gut microbiome, yet was adequate to boost antitumor immunity and synergize with ICB. These findings echoed the increasingly noted roles of ileal microbiome in dictating the immune state and governing the therapeutic efficacy in colon cancer patients,^[^ ^8^, ^10^ ^]^ and call for the attention to the varied intestinal microbial composition across different intestinal tract segments.

The ileum preference of chemotherapy likely stems from at least two major reasons. First, TACs in the ileum exhibit much faster proliferative rates than TACs in the colon, making them more susceptible to chemotherapeutic drugs (Figure 1I). At a relatively low dose, chemotherapeutic drugs cause ileum‐restricted DNA damage, thus avoiding the broad disruption of gut homeostasis often occurring in clinical treatment. Second, the unique cytoarchitecture of the ileal crypt, with multiple types of cells all located in close proximity to the crypt base, enables the local interplay among the TACs, Th1 cells and especially Paneth cells residing exclusively in the crypts of the small intestine. This compartmental cell‐cell interplay functions as a transducer to shape the indigenous microbial composition restricted in the ileum. Recently, chemotherapeutic drugs have been widely tested in clinical trials to combine with ICB for their capability to stimulate antitumor immunity. Of note, most of these trials have adopted a classical MTD chemotherapy regimen, which is expected to impose extensive effects, including immune suppression as well as the gastrointestinal and systemic toxicities, adding uncertainties to patient outcomes.^[^ ^49^, ^50^, ^51^ ^]^ In these circumstances, a global dysbiosis and the decreased microbial diversity have been concurrently observed.^[^ ^52^ ^]^ According to our findings, a low‐dose regimen (ranging from 1/20 to 1/10 MTD) could avoid extensive DNA damage along the whole gut, and from the perspective of spatial microbiome modulation, it is advantageous in the combination with ICB therapy.^[^ ^53^ ^]^

In contrast to the large intestinal microbiota, small intestinal bacteria exhibit significantly lower richness and diversity. The ileal mucosa is relatively sterile due to several restrictive factors coexisting in this area, such as the relatively higher oxygen content, the continuously secreted mucus and the abundant antimicrobial peptides. According to our results, *SFB* and *Lactobacillus* are the most affected bacterial in the ileum, which could be explained by their colonization preference for the small intestine, especially in the terminal ileum. It remains unclear how *SFB* overgrowth together with the *Lactobacillus* shrinkage in ileal regions synergizes with the ICB therapy. It is plausible that the proinflammatory effect of *SFB* ^[^ ^25^, ^28^ ^]^ accompanied by a compromised anti‐inflammatory role of *Lactobacillus* ^[^ ^29^, ^30^ ^]^ could shape a poised systemic immune state that renders the tumor immune microenvironment responsive to ICB therapy. Alternatively, drug‐induced reversal of their relative abundance could rewire the local microbial metabolic profile, and, due to the larger surface area and the thinner mucus layer of the ileum compared to the colon, the metabolites could be readily absorbed into the circulation and remodel the remote tumor immunity. One limitation of our study is that we did not prove the direct contribution of either *SFB* outgrowth or *Lactobacillus* depletion to the therapeutic efficacy, due to technical limitations. *SFB* culture is technically difficult,^[^ ^31^ ^]^ making it highly demanding to generate an *SFB* mono‐colonized mouse model. Transplantation of the *SFB*‐containing microbiome from TAC mice into *SFB*‐deficient JAX mice could be an alternative tool,^[^ ^25^ ^]^ yet the required mouse sources are not available in China. Moreover, approaches for the ileum‐specific depletion of *Lactobacillus* are not available. Instead, we took an alternative approach of systemic or colon‐specific microbiome depletion in parallel. Only systemic depletion abolished the therapeutic benefits in boosting antitumor immunity (Figure 2F,H), which emphasizes a vital role of the ileal microbial alteration, characterized by the dominance of *SFB* and the depletion of *Lactobacillus*.

Our findings also complement the previous findings by demonstrating the intestinal AIM2 inflammasome plays a double‐faced role in either rendering antitumor immunity or causing severe enteritis in response to chemotherapy. Previous studies have discovered that the AIM2 inflammasome is required for chemotherapy‐induced intestinal injury and diarrhea.^[^ ^12^ ^]^ In this study, AIM2 inflammasome activation triggered cell‐cell crosstalk confined to the proximity of the ileal crypt, resulting in a therapeutic synergy with ICB without the severe inflammation and tissue damage. The discrepancy is, at least in part, due to the location of AIM2 activation, which depends on the drug dosages. Under a toxic dosage associated with severe intestinal damage, antigen‐presenting cells (APCs) engulf dsDNA‐containing exosomes released from a mass of apoptotic enterocytes, causing the activation of AIM2 in APCs. In contrast, at low doses, AIM2 activation is restricted to ileal epithelial cells rather than submucosal APCs, thus avoiding the severe inflammation and tissue damage.

Together, our findings may advance the field in several aspects. First, we have demonstrated the spatial preference of low‐dose chemotherapy for the ileal microbiome and highlighted the translational value of low‐dose chemotherapy to boost antitumor immunity. Second, we provide the molecular links between the chemotherapy‐induced genomic stress in intestinal epithelial cells and microbiota alteration, demonstrating the role of the DNA sensor AIM2 in linking the genomic stress to the remodeled microbiota. Third, we provide an interaction map depicting the spatial interplay among ileal epithelial cells, immune cells and local bacteria, gaining insights into compartmentalized cell crosstalk in the proximity of ileal crypts for the spatial regulation of the gut microbiome.

## 4 Experimental Section

### Study Design

The main objectives of this study were to elucidate how low‐dose chemotherapy spatially shapes gut microbiome, to determine whether it synergizes with ICB therapy to retard tumor progression, and to assess the molecular basis linking the chemotherapy‐induced genomic stress to indigenous bacteria change. Mice with different genetic backgrounds were used to evaluate drug caused intestinal toxicity and tumor response, with approved humane end points to terminate in vivo experiments. Mice tissues (microbiome, gut, and tumor grafts) were harvested for histopathology and ex vivo immune functional profiling studies (tumor infiltrated immune cells, intestinal immune cells, small intestinal organoids). Mice were randomly assigned to the experimental groups and studies were not blinded. The number of mice used in each experiment is indicated in figure legends.

### Mice

All studies and procedures involving animal subjects were performed under the approval of the Institutional Animal Care and Use Committee (IACUC) at Shanghai Institute of Materia Medica (2022‐06‐GMY‐26, 2023‐01‐HM‐01), C57BL/6 mice and BALB/c nude mice (6–8 weeks old, female) were obtained from Beijing Vital River Laboratory Animal Technology. Chinese Academy of Sciences. *Aim2* ^−/−^ mice were kindly gifted by Prof. Bing Sun (Shanghai Institute of Biochemistry and Cell Biology, Chinese Academy of Sciences). Littermate WT mice were used in all studies concerning *Aim2* ^−/−^ mice. Lgr5‐EGFP‐IRES‐creERT2 mice were obtained from the Jackson Laboratory. Intestinal epithelial cell‐specific AIM2 knockout mice (*Aim2^flox/flox^;Villin‐Cre*) were generated by breeding mice carrying a conditional *Aim2* allele (*Aim2^flox/flox^*, obtained from Gempharmatech Co., Ltd, Jiangsu, China) with mice expressing Cre in the intestine (*Villin‐Cre*, obtained from Gempharmatech. For genotyping of *Aim2^−/−^* mice, RT‐PCR was performed with RNA extracted from blood using primers: F: 5′‐CAC ACT CGA CGT GGC AGA TAG GAC‐3′. R1: 5′‐CAG CAC CGT GAC AAC AAG TGG‐3′. R2: 5′‐ TCG ATG CGA TCT GCG TTC TTC, resulting in 380 and 230 bp band for wildtype allele and 340 and 230 bands for AIM2‐knockout allele. Genotyping of *Aim2^flox/flox^* mice was performed with genomic DNA extracted from tail and following primers F: 5′‐CTT GCC AAT GTA CTC AAC AGT TCT C‐3′ and R: 5′‐AGA AGG TAA CAG AGG TGC CAT CTA G‐3′, resulting a 367 bp band for conditional allele and a 263 bp band for the wildtype allele. Genotyping the *Villin‐Cre* mice was performed with genomic DNA extracted from tail and following primers F: 5′‐GGG CAG TCT GGT ACT TCC AAG CT‐3′, R: 5′‐AGT TTC CAA ACT CCA GGT GAC AGG‐3′, resulting a 383 bp band. All mice were housed in an animal facility and maintained in a temperature and light controlled environment with an alternating 12 h light/dark cycle.

### Cell Lines

MC38 cell line (syngeneic from C57BL/6 mice) was kindly gifted by Prof. Yong Cang (ShanghaiTech University, Shanghai, China). HEK 293T cell line was purchased from the American Type Culture Collection (ATCC, Manassas, USA). Cells were cultured in DMEM medium containing 10% FBS. All reagents were purchased from Gibco‐Invitrogen. All cell lines were cultured at 37 °C with 5% CO_2_ and regularly tested to be free of mycoplasma contamination.

### Cell Transfection

Murine *Aim2* or *Asc* CDS sequences were cloned into pCMV6‐Entry Mammalian Expression Vector (Origene). HEK 293T cells were transiently transfected with AIM2/ASC or ASC plasmids using Lipofectamine 3000 transfection reagent (#L30000015, Invitrogen) according to the manufacturer's instructions.

### Immunofluorescence

Cells were seeded on glass coverslips, fixed with 4% paraformaldehyde and permeabilized with PBS containing 0.2% Triton‐X 100. After blocking with 3% BSA for 1 h, cells were incubated with anti‐dsDNA (#27 156, Abcam) or anti‐ASC (#67 824, Cell Signaling Technology) antibodies. After incubation with the secondary antibodies, cells were stained with DAPI and mounted with ProLong Diamond Antifade Mountant (Invitrogen). Images were taken on Lecia TCS SPS CFSMP microscope.

### MC38 Tumor Models

MC38 colon adenocarcinoma cells (5 × 10^5^) were subcutaneously injected into the flank of syngeneic C57BL/6 or BALB/c nude mice. Mice were treated when tumors reached 50–80 mm^3^ in size. Tumor growth was monitored by the measurement of tumor size using calipers once every 3 days using the formula (length × width^2^)/2.

### Animal Treatment

Regimen of CPT‐11 treatment was slightly different according to purpose of the study. In brief, to determine the intestinal toxicity, mice were treated with gradient doses of CPT‐11 (#MB1126, Meilunbio, 10–90 mg kg^−1^, i.p.) for 10 consecutive days. Body weight was measured daily and intestine length was measured after mice were sacrificed. To examine the impact of CPT‐11 on the microbiota and intestinal immunity, mice were treated with CPT‐11 (20 or 90 mg kg^−1^, i.p.) for 7 consecutive days. To observe the therapeutic effect of CPT‐11 in combination with anti‐PD1 antibody, mice were treated with CPT‐11 (20 mg kg^−1^, i.p.) daily for two weeks. For the sequential treatment experiment, mice were treated with CPT‐11 (20 mg kg^−1^, i.p.) for 7 consecutive days in the first or the second week of the experiment.

For IL‐18 or IFN‐γ blockage experiments, mice were pre‐treated with anti‐IL‐18 (#BE0237, BioXcell, 5 mg kg^−1^, i.p.) or anti‐IFN‐γ (#BE0055, BioXcell, 5 mg kg^−1^, i.p.) antibodies for 2 consecutive days followed by continuous treatment once every 3 days throughout the experiment. For JAK1/2 inhibition in vivo, mice were treated with INCB018424 (#MB5455, Meilunbio, 100 mg kg^−1^, p.o.) alone or in combination with CPT‐11 (20 mg kg^−1^, i.p.) or vehicle for 7 consecutive days. For anti‐PD‐1 treatment, mice were treated with anti‐PD1 antibody (#BE0146, BioXcell, 5 mg kg^−1^, i.p.) twice per week. In the combination with CPT‐11, anti‐PD1 treatment was started 2 days after the first CPT‐11 dosage and was continuously treated twice per week throughout the experiment.

### Antibiotic Treatments

For specific deletion of colon microbiota, mice were treated by enema with 500 µl ABX solution containing neomycin (#MB1716, Meilunbio, 2 g L^−1^), vancomycin (#MB1260, Meilunbio, 1 g L^−1^), and imipenem (#MB1748, Meilunbio, 0.5 g L^−1^) twice a day for 2 weeks.

For depletion of microbiota along the whole gut, mice were treated with an ABX solution containing neomycin (#MB1716, Meilunbio, 1 g L^−1^), vancomycin (#MB1260, Meilunbio, 0.5 g L^−1^), and imipenem (#MB1748, Meilunbio, 0.25 g L^−1^). ABX was dissolved into the sterile drinking water. Solutions and bottles were changed twice a week. Mice were treated for two weeks before tumor implantation and were continuously treated throughout the experiment.

### Intestinal Permeability Assay

Mice were fasted for 4–6 h prior to the test. After fasting, mice were orally administrated with 150 µl per mouse of 80 mg mL^−1^ 4 kDa FITC‐dextran (#FD4‐1G, Sigma) in sterile PBS or with vehicle control. Four hours later, blood samples were collected and centrifuged at 2000 g for 15 min to obtain plasma and then transferred to a black opaque‐bottom 96‐well plate. Fluorescence was determined at 530 nm with excitation at 485 nm.

### Immunohistochemistry

Immunohistochemical analysis was performed by Shanghai Zuocheng Biological Technology. For Ki67, γH2AX, ZO‐1, and lysozyme staining, ileal or colon tissues from mice were fixed with 4% paraformaldehyde and embedded in paraffin. Tissue sections were then deparaffinized and rehydrated, followed by antigen retrieval via pre‐treating sections with citrate buffer (pH 6.0) for 30 min in a microwave (700 W) and then for another 30 min at room temperature (RT). Samples were then blocked with 5% BSA for 1 h at RT. The primary antibodies for Ki67 (#ab16667, Abcam,), γH2AX (Ser139) (#9718, Cell Signaling Technology), ZO‐1 (#61‐7300, Invitrogen), and lysozyme (#NBP2‐67507, Novus) were incubated overnight, followed by the secondary antibody (A‐11008, Invitrogen) incubation for 1 h at RT. Images were captured with Lecia TCS SPS CFSMP microscope. For TUNEL staining, In Situ Cell Death Detection Kit (#11 684 817 910, Roche) was used according to the manufacturer's instructions. Images were captured with NanoZoomer S210 (Hamamatsu, Japan) and analyzed using NDP.View 2 software.

For Paneth cell quantification, the number of Alcian blue positive cells in each ileal crypt base was counted and the average in each sample was calculated.

### 16S rRNA Sequencing and Microbial Composition Analysis

Bacterial 16S rRNA gene sequencing was performed by Majorbio Bio‐Pharm Technology (Shanghai China). Briefly, total microbial genomic DNA was extracted from colon and ileum tissues using the E.Z.N.A. Soil DNA Kit (Omega Bio‐tek) according to the manufacturer's instructions. The hypervariable region of V3‐V4 of the bacterial 16S rRNA gene was amplified with primer pairs 338F (5′‐ACT CCT ACG GGA GGC AGC AG‐3′) and 806R (5′‐GGA CTA CHV GGG TWT CTA AT‐3′). Purified amplicons were pooled in equimolar amounts and paired‐end sequenced on an Illumina MiSeq PE300 platform/NovaSeq PE250 platform (Illumina, San Diego, USA). Raw FASTQ files were de‐multiplexed using an in‐house perl script, and then quality‐filtered by fastp version 0.19.6^[^ ^54^ ^]^ and merged by FLASH version 1.2.7.^[^ ^55^ ^]^ Then the optimized sequences were clustered into operational taxonomic units (OTUs) using UPARSE 7.1^[^ ^56^ ^]^ with 97% sequence similarity level. The most abundant sequence for each OTU was selected as a representative sequence.

The taxonomy of each OTU representative sequence was analyzed by RDP Classifier version 2.2^[^ ^57^ ^]^ against the 16S rRNA gene database (eg. Silva v138) using a confidence threshold of 0.7. Bioinformatic analysis of the gut microbiota was carried out using the Majorbio Cloud platform (https://cloud.majorbio.com). The similarity among the microbial communities in different samples was determined by principal coordinate analysis (PCoA) based on Bray‐curtis dissimilarity using Vegan v2.5‐3 package. The linear discriminant analysis (LDA) effect size (LEfSe)^[^ ^58^ ^]^ (http://huttenhower.sph.harvard.edu/LEfSe) was performed to identify the significantly abundant taxa (phylum to genera) of bacteria among the different groups (LDA score > 4, *p* < 0.05).

### Isolation of Single Ileal Crypt Cells

Mice ileal crypts were isolated as previously described with slight modification.^[^ ^59^, ^60^ ^]^ In brief, mice ileum was dissected, flushed, opened and incubated in cold PBS containing 20 mm EDTA for 90 min followed by vigorous shaking. The supernatant was the villous fraction and was discarded. The sediment was re‐incubated with EDTA for 30 min on ice followed by shaking. The supernatant was filtered through a 70‐µm strainer and was examined by microscopy to make sure that crypts were essentially enriched. After that, the supernatant was centrifuged at 350 g, 4 °C for 5 min to collect crypts. Isolated crypts were then incubated in 1 mg ml^−1^ Dispase II (#40104ES80, Yeasen) at 37 °C for 10 min to obtain single cells.

### RNA‐Sequencing Analysis

RNA‐sequencing analysis was performed by Majorbio Bio‐Pharm Technology. Gene expression fold change and adjusted p‐value between vehicle and CPT‐11 groups were obtained using Deseq2. For pathway enrichment analysis, 131 genes up‐regulated by CPT‐11 treatment (cutoff: fold change > 2, adjusted *p*‐value < 0.01) were analyzed using hallmark gene sets extracted from molecular signatures database (MSigDB). Gene Set Enrichment Analysis (GSEA) analysis was performed using the Paneth cell signature gene sets.^[^ ^44^ ^]^ GSEA and Pathway enrichment analysis was performed using ClusterProfiler on R 4.0.2.

### Flow Cytometry Analysis

For the isolation of tumor infiltrating lymphocytes (TILs), tumors were excised and minced, followed by incubation in 1640 RPMI medium with collagenase IV (#LS004188, Worthington, 1 mg ml^−1^) and DNase I (#10 104 159 001, Roche, 0.25 mg ml^−1^) at 37 °C for 35 min. The mixture was then triturated and filtered through a 70‐µm strainer, and centrifuged at 350 g, 4 °C for 5 min. Single cells were collected and passed through a 70‐µm strainer followed by lysis of red blood cells.

For isolation of lamina propria lymphocytes (LPL), mice ileal tissues were finely removed from adipose tissues and Peyer patch. Ileum was cut into pieces, followed by the incubation in PBS buffer containing 5% FBS, 15 mm HEPES and 5 mm EDTA at 37 °C for 30 min. Intraepithelial lymphocytes were removed by vigorous shaking and remaining tissues were digested in DMEM containing 10% FBS, 15 mm HEPES (Sigma‐Aldrich), collagenase IV (#LS004188, Worthington, 1 mg ml^−1^), and DNase I (#10 104 159 001, Roche, 0.25 mg ml^−1^) at 37 °C for 1 h. Washed cells were then suspended in Percoll and centrifuged at 1260 g for 30 min to enrich LPL.

For single cell preparation for mesenteric lymph nodes (mLN), mLN were finely removed from adipose tissues, triturated and filtered through a 70‐µm strainer, followed by centrifuging at 350 g, 4 °C for 5 min.

For flow cytometry analysis, single cells were incubated with FLICA‐YVAD (#9122, ImmunoChemistry Technologies) for 1 h at 37 °C and re‐suspended every 20 min, followed by washing according to the manufacturer's instructions. Single cells were stained with Fixable Viability Stain 510 (#564 406, BD) and incubated with Fc block (#553 141, BD) on ice for 15 min to prevent non‐specific binding, followed by washing and staining for surface markers in antibody mixture on ice for 30 min. For intracellular staining, cells were incubated in 1640 RPMI medium with 10% FBS and 1 × Cell Stimulation Cocktail (#00‐4975‐03, Invitrogen) for 4 h in a humidified 37 °C 5% CO_2_ incubator, followed by staining for surface markers. Cells were then fixed, permeabilized and stained for intracellular molecules. Antibodies (and clones) used were as follows: FITC‐conjugated anti‐CD45 (clone 30‐F11, BioLegend), APC/Cyanine7‐conjugated anti‐CD3 (clone 17A2, BioLegend), PerCP/Cyaneine5.5‐conjugated anti‐CD4 (clone GK1.5, BioLegend), BV421‐conjugated anti‐CD4 (clone GK1.5, BioLegend), PerCP/Cyaneine5.5‐conjugated anti‐CD8 (clone 53–6.7, eBioscience), PE‐conjugated anti‐FOXP3 (NRRF‐30, eBioscience), APC‐conjugated anti‐T‐bet (4B10, BioLegend), PE‐conjugated anti‐IL‐17A (eBio17B7, 12‐7177‐81), APC‐conjugated anti‐IFN‐γ (XMG1.2, eBioscience), and PE/Cyanine7‐conjugated TNF‐α (clone MP6‐XT22, BioLegend). Data were recorded on LSRFortessa X‐20 (BD Biosciences) for analysis using Flowjo.

### Enzyme‐Linked Immunosorbent Assay (ELISA)

Mice ileum tissues (0.2 g) were transferred into 0.2 ml cold homogenate buffer (PBS with protease inhibitor cocktail (#5871s, Cell Signaling Technology)) and homogenized. The homogenate was centrifuged at 11 000 g, 4 °C for 10 min and the supernatant was collected. Mouse IL‐18 ELISA Kit (Abcam, ab216165), Mouse IL‐1β beta ELISA Kit (abs520001‐96T) and Mouse IFN‐γ ELISA Kit (BioLegend) were used according to the manufacturer's instructions.

### Immunoblotting Assay

Ileum tissues were lysed in 2% SDS buffer‐containing protease inhibitor cocktail. After centrifuging at 11 000 g, 4 °C for 10 min, the supernatant was obtained for SDS‐PAGE. Proteins were then transferred to nitrocellulose membranes, followed by blockage with 3% BSA for 1 h at RT. Membranes were incubated with primary antibodies overnight at 4 °C. The next day, membranes were washed, and then incubated with HRP‐conjugated secondary antibodies at RT for 1 h. Antibodies used in this study included anti‐cleaved caspase‐1 (#89 332, Cell Signaling Technology), anti‐lysozyme (#NBP2‐67507, Novus), anti‐β‐actin (#3700s, Cell Signaling Technology), and anti‐GAPDH (#60004‐1‐lg, Proteintech).

### Quantitative PCR (qPCR) for Ileal *SFB* and *Lactobacillus* Analysis

To quantify bacterial load of *SFB* and *Lactobacillus* in the ileum, genomic DNA was extracted from the same ileum samples with equivalent length using Fast DNA Stool Mini Kit (Qiagen) according to the manufacturer's instructions. qPCR was conducted on samples to determine the relative amount of *SFB* and *Lactobacillus* in the ileum, normalized by murine Gapdh as an internal control. Primer sequences were as follows (5′‐3′)

*SFB* fwd AGG AGG AGT CTG CGG CAC ATT AGC, rev TCC CCA CTG CTG CCT CCC GTA G.

*L. Johnsonii* fwd CAC TAG ACG CAT GTC TAG AG, rev AGT CTC TCA ACT CGG CTA TG.

*L. Murinus* fwd TCG AAC GAA ACT TCT TTA TCA CC, rev CGT TCG CCA CTC AAC TCT TT.

*L. Reuteri* fwd ACC GAG AAC ACC GCG TTA TTT, rev ACC TAA ACA ATC AAA GAT TGT CT.

Mouse *Gapdh* fwd CAT CAC TGC CAC CCA GAA GAC TG, rev ATG CCA GTG AGC TTC CCG TTC AG.

### Fluorescence In Situ Hybridization (FISH) Analysis

Ribosomal RNA (rRNA) FISH was performed for the visualization of bacteria in the ileum. The protocol was adapted from Leore et al.^[^ ^61^ ^]^ Briefly, Carnoy‐fixed sections of the ileum were kept at 4 °C for at least 2 h. Samples were then incubated in a solution containing 7 mg ml^−1^ of lysozyme in working buffer (0.1 m Tris‐HCl‐5 mm EDTA, pH 7.2) for 10 min at 37 °C, in 2 × SSC buffer (#AM9770, Invitrogen) for 10 min at RT, and then treated with 10 µg ml^−1^ proteinase‐K (#AM2548, Invitrogen) for 10 min at RT. Samples were then incubated twice with 2 × SSC buffer for 5 min at RT, rinsed with a wash buffer containing 25% formamide, 2 × SSC buffer and nuclease‐free water, and then incubated with fresh wash buffer for an additional 5 min at RT. Probes (EUB338: FAM‐5′ GCT GCC TCC CGT AGG AGT −3′ and SFB: Cy3‐5′ GGG TAC TTA TTG CGT TTG CGA CGG CAC −3′)^[^ ^62^ ^]^ were hybridized to the tissue overnight at 37 °C, using a hybridization buffer containing 25% formamide, 10% dextran sulfate, 1 mg ml^−1^ Yeast tRNA (#AM7119, Ambion), 2 × SSC buffer, 0.02% BSA and nuclease‐free water. Unbound probes were removed by rinsing samples in wash buffer for 30 min at 37 °C. Nuclear DNA was stained by incubation with DAPI for 30 min at RT. Slides were imaged using Lecia TCS SPS CFSMP microscope.

For *Defa1* mRNA FISH, ileum paraffin sections were hybridized with *Defa1*‐specific fluorescence RNA probe mixes (FAM‐5′‐GTC CTC TTC TCC TGG CTG CTC CTC A‐3′, FAM‐5′‐ ATA CGG CCT GGT CCT CTT CTC CTG G‐3′, FAM‐5′‐CAA TAG CAT ACC AGA TCT CTC AAC G‐ 3′), using RNA FISH Kit (GenePharma) according to the manufacturer's instructions.

### Organoid Culture and Treatment

Small intestinal crypts were isolated and cultured in standard culture medium (advanced DMEM/F12 containing 100 ng ml^−1^ EGF, 100 ng ml^−1^ Noggin, 100 ng ml^−1^ R‐spondin1, 1 × B‐27, 1 × N2 and 1 mm N‐Acetylcysteine) as previously described with slight modifications.^[^ ^46^ ^]^ Organoids were treated for recombinant murine IFN‐γ (0.1–10 ng ml^−1^) and INCB018424 (5 µm) as indicated. For RT‐qPCR, organoids were seeded in 24‐well‐pate at 200 crypt per well. For propidium iodide staining and live imaging, organoids were seeded in 8‐well chamber‐slide at 60 crypt per well. Images were acquired using inverted microscope.

### Transmission Electron Microscopy (TEM) Analysis

Ileal tissues were immersed in 1.5% glutaraldehyde fixative, washed with cacodylate buffer and transferred to a 1% OsO_4_ fixative solution for fixation. After dehydration and embedding in Epon, ultrathin sections were cut and analyzed using Hitachi HT‐7800 TEM.

### Quantification of dsDNA

Peritoneal lavage fluid was harvested using 1 ml sterile PBS injected into peritoneum. Flush fluid from 3‐cm lengths of the ileum was harvested using 0.5 ml PBS. The fluid was subsequently centrifuged at 5 000 rpm for 5 min, and the supernatants were centrifuged again. The second supernatants correspond to cell‐depleted samples. dsDNA in the supernatants was quantitated using the PicoGreen assay (Life Technologies).

### RT‐qPCR

RNA was extracted with TRIzol reagent. Total RNA (1 µg) was used for cDNA synthesis using the HiScript II Q Select RT SuperMix for qPCR (#R233, Vazyme) according to the manufacturer's instructions. RT‐qPCR was performed in a thermal cycler for qPCR (Bio‐Rad) with SYBR qPCR Master Mix (#Q711, Vazyme). All samples were analyzed in duplicate and the quality was checked by melting curve. All the qPCR data were normalized by that of HPRT. Primer sequences were as follows (5′‐3′):

Mouse *Hprt* fwd TGC TCG AGA TGT CAT GAA GGA G, rev CAG AGG GCC ACA ATG TGA TG

Mouse *Defa1* fwd TCA AGA GGC TGC AAA GGA AGA GAA C, rev TGG TCT CCA TGT TCA GCG ACA GC

Mouse *Defa2* fwd CCA GGC TGA TCC TAT CCA AA, rev GTC CCA TTC ATG CGT TCT CT

Mouse *Defa29* fwd CAC CAC CCA AGC TCC AAA TAC ACA G, rev ATC GTG AGG ACC AAA AGC AAA TGG

Mouse *Defa6* fwd CCT TCC AGG TCC AGG CTG AT, rev TGA GAA GTG GTC ATC AGG CAC

Mouse *Ifng* fwd GCT TTG CAG CTC TTC CTC AT, rev CCA GTT CCT CCA GAT ATC CAA G

### Statistics

Data were expressed as means ± SEM. Statistical significance was determined using unpaired two‐tailed Student's t‐test. Log‐rank test was conducted for survival analysis. Differences were considered to be statistically significant at *p* < 0.05. Statistical calculations were performed with GraphPad Prism.

## Conflict of Interest

The authors declare no conflict of interest.

## Author Contributions

C.P., Y.L., and Y.F. contributed equally to this work. M.H. conceived the project and supervised the study; M.G., J.X., and J.D. performed the supervision; C.P., J.X., Y.L., and Y.F. designed the experiments, performed the research, analyzed the data and prepared the figures; Y.Y., S.T., S.T., X.G., Z.D., Z.G., Y.L., S.C., and T.W. performed part of experiments or provided technical assistance; M.H., C.P., J.X., and Y.L. wrote the manuscript. All authors approved the final version of the manuscript.

## Supporting information

Supporting Information

## Data Availability

The data that support the findings of this study are available from the corresponding author upon reasonable request.
