# Supplementary material for: Low‐Dose Chemotherapy Preferentially Shapes the Ileal Microbiome and Augments the Response to Immune Checkpoint Blockade by Activating AIM2 Inflammasome in Ileal Epithelial Cells
Source: Adv Sci (Weinh). 2024 Jan 8;11(11):2304781. doi: 10.1002/advs.202304781 (PMC10953579; doi:10.1002/advs.202304781)
Supplement: Supplementary file 1 — Supporting Information [file ADVS-11-2304781-s001.pdf]

ADVANCED SCIENCE
Open Access

Supporting Information

for *Adv. Sci.*, DOI 10.1002/advs.202304781

Low-Dose Chemotherapy Preferentially Shapes the Ileal Microbiome and Augments the Response to Immune Checkpoint Blockade by Activating AIM2 Inflammasome in Ileal Epithelial Cells

Congying Pu, Yize Li, Yixian Fu, Yiyang Yan, Siyao Tao, Shuai Tang, Xiameng Gai, Ziyi Ding, Zhenjie Gan, Yingluo Liu, Siyuwei Cao, Ting Wang, Jian Ding, Jun Xu, Meiyu Geng* and Min Huang**

Supporting information

Low-dose chemotherapy preferentially shapes the ileal microbiome and augments the response to immune checkpoint blockade by activating AIM2 inflammasome in ileal epithelial cells

Authors: *Congying Pu, Yize Li, Yixian Fu, Yiyang Yan, Siyao Tao, Shuai Tang, Xiameng Gai, Ziyi Ding, Zhenjie Gan, Yingluo Liu, Siyuwei Cao, Ting Wang, Jian Ding, Jun Xu*, Meiyu Geng*, Min Huang**

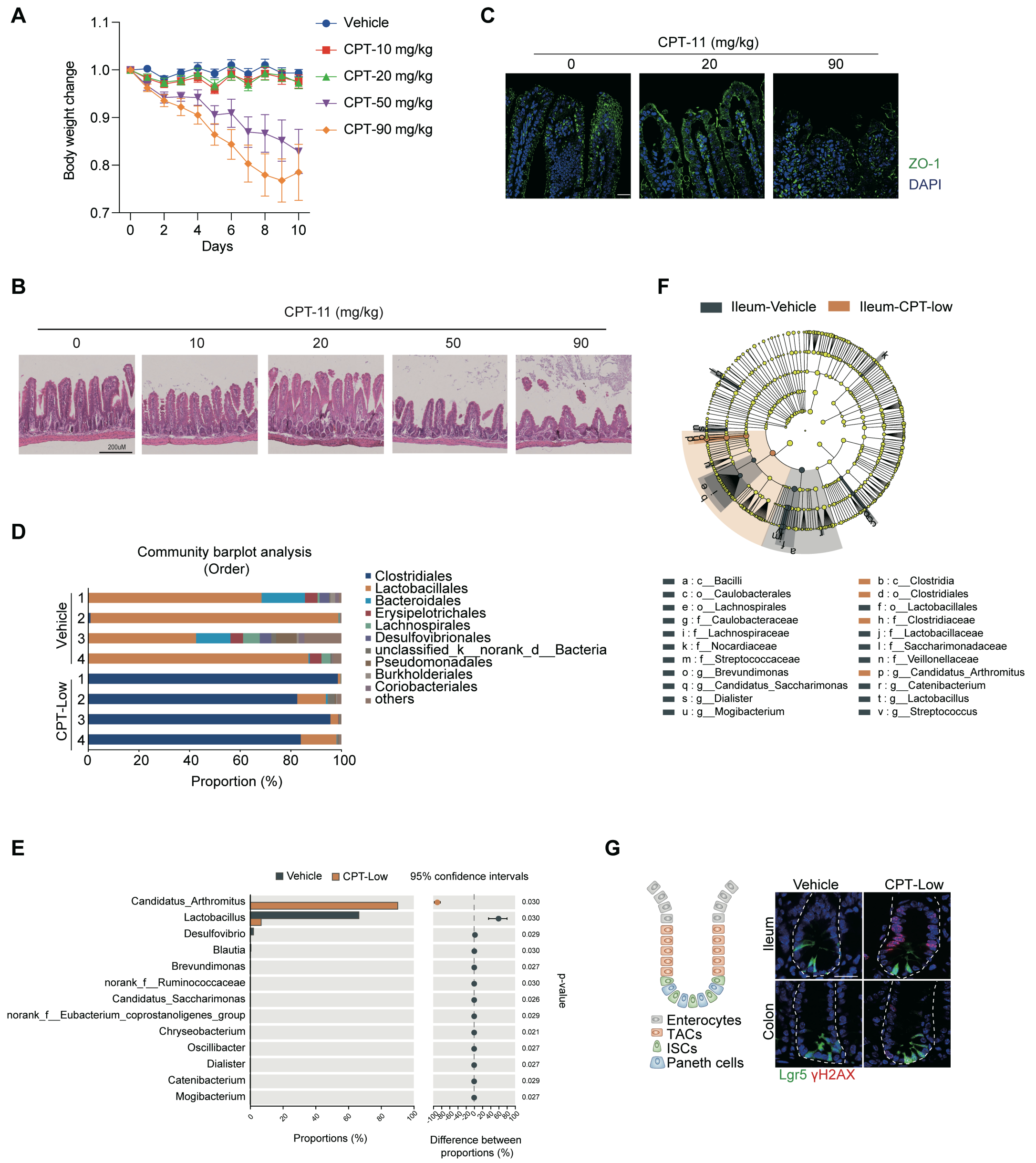

Figure S1. Low-dose chemotherapy remodels ileal microbiome without causing the intestinal toxicity.

(A-B) Dose-response of CPT-11-caused intestinal toxicity. C57BL/6 mice were treated with different doses of CPT-11 or vehicle control as indicated for 10 consecutive days (n = 6 or 7 per group). **(A)** Mice body weight curve. **(B)** Representative images of immunohistochemical staining of ileum tissues.

(C) Representative images of immunohistofluorescent staining of ZO-1 in the ileum. C57BL/6 mice were treated with CPT-Low (20 mg/kg), CPT-Toxic (90 mg/kg) or vehicle control for 7 consecutive days.

(D-F) The impact of CPT-Low on the ileal microbiome. C57BL/6 mice were treated with CPT-Low (20 mg/kg) or vehicle control for 7 consecutive days (n = 4 per group) and ileum tissues were collected for 16S rRNA gene sequencing. **(D)** Relative proportion of ileal microbiota at the order

level. **(E)** Genus level comparison of ileal microbiota. **(F)** Taxonomic cladogram from LEfSe showing significant differences in taxa. Dot size is proportional to the abundance of the taxon.
(G) CPT-caused DNA damage in the ileum. Lgr5-EGFP mice were treated with CPT-Low (20 mg/kg) or vehicle control for 6 h (n = 3 per group) and ileum tissues were collected for γH2AX staining. Left, a schematic illustration of the cytoarchitecture of the ileal crypt with different cell lineages. TACs, transit amplifying cells. ISCs, intestinal stem cells. Right, representative images of immunohistofluorescent staining of γH2AX.
Data were represented as mean ± SEM. p values were calculated by two-tailed Student's *t*-test.

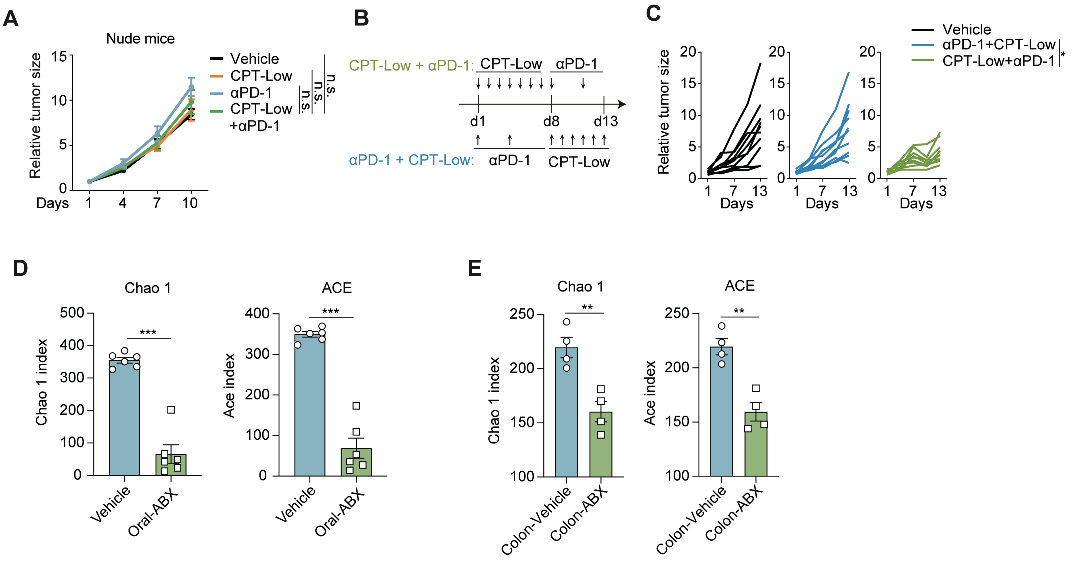

Figure S2. Ileum-specific microbiome alteration activates the extraintestinal antitumor immunity and augments the response to anti-PD1 therapy.

(A) Tumor growth curve in nude mice. BALB/c nude mice bearing MC38 tumors were treated with anti-PD1 antibody (5 mg/kg, twice per week) alone or in combination with CPT-Low (20 mg/kg, daily) (n = 7 or 8 per group).

(B, C) Sequential treatment of CPT-Low and anti-PD1 antibody. C57BL/6 mice bearing MC38 tumors were treated with anti-PD-1 antibody (5 mg/kg, twice per week) or CPT-Low (20 mg/kg, daily) (n = 8 or 10 per group). **(B)** Scheme showing the treatment procedure. **(C)** Tumor growth curve of individual mouse.

(D, E) Alpha diversity of the overall gut microbiome shown by Chao 1 and ACE index. Feces from oral-ABX (**D,** n = 6 per group) or colon-ABX (**E,** n = 4 per group) treated mice were collected for 16S rRNA gene sequencing.

Data were represented as mean ± SEM. p values were calculated by two-tailed Student's *t*-test. n.s., not significant; $^{*}p < 0.05$; $^{**}p < 0.01$; $^{***}p < 0.001$.

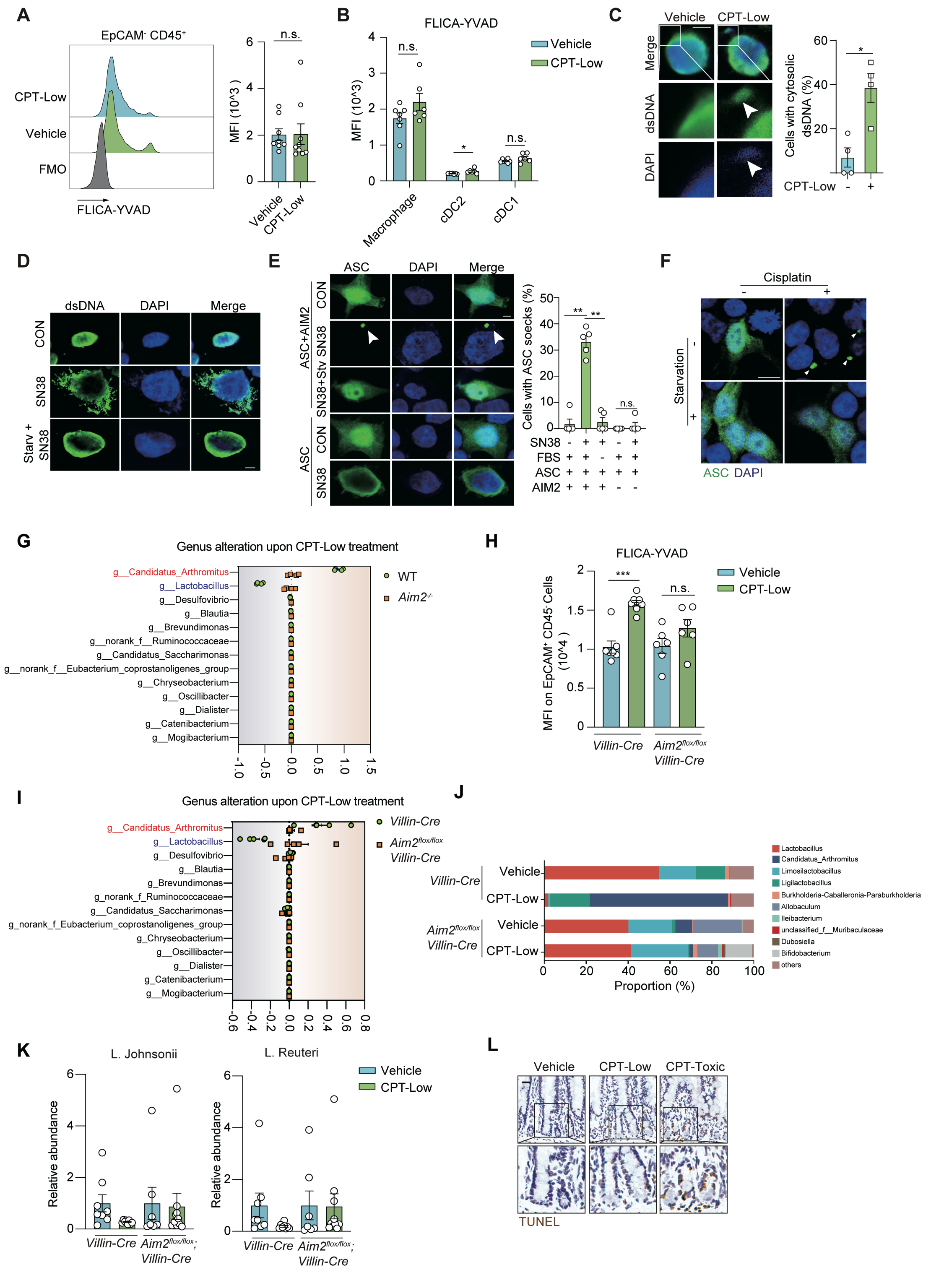

Figure S3. Chemotherapy causes DNA damage and activates AIM2 inflammasome in proliferative cells.

(A-B) Caspase-1 activity in ileal epithelial cells. C57BL/6 mice were treated with low-dose CPT-11 (20 mg/kg) or vehicle control for 7 consecutive days. Caspase-1 activity was assessed by a specific probe FLICA-YVAD. Shown are representative flow cytometry histogram and relative mean fluorescence intensity (MFI). Fluorescence minus one (FMO) control was used to define negative

staining for FLICA probe. **(A)** $EpCAM^{-}CD45^{+}$ intraepithelial lymphocytes (IEL) from ileal crypts.
(B) Macrophage, cDC1 and cCD2 from lamina propria.

(C) Cytosolic dsDNA production in ileal crypt cells. Mice were treated as in (A) followed by ileal crypt cell isolation and dsDNA staining (n = 4 per group). Inset depicts a higher magnification field. Cells with cytosolic dsDNA staining were counted and normalized by total cell counts. Arrows indicate cytosolic dsDNA. Scale bar, 5 μm

(D) Representative images of immunofluorescent staining of dsDNA. HEK 293T cells were exposed to serum starvation (Starv) for 24 h followed by treatment of SN38 (100 nM) or vehicle control for another 24 h.

(E, F) Representative images of immunofluorescent staining of ASC and quantification of ASC specks. **(E)** HEK 293T cells transfected with ASC alone or co-transfected with ASC and AIM2 were treated as in (D). Arrows indicate ASC specks. Scale bar, 5 μm. ASC speck-containing cells were counted and normalized by the total cell counts. **(F)** HEK 293T cells co-transfected with ASC and AIM2 were starved for 24 h followed by cisplatin (1 μM) treatment for another 24 h.

(G) Alteration of top-10 abundant microbiota at the genus level. *Aim2*$^{-/-}$ and littermate wildtype (WT) mice were treated as in (A) (n = 4 per group) and ileum tissues were harvested for 16S rRNA gene sequencing.

(H) Caspase-1 activity in intestinal epithelium-specific AIM2 knockout mice. *Aim2*$^{flox/flox}$*;Villin-Cre* or littermate control mice (*Villin-Cre*) were treated with low-dose CPT-11 (20 mg/kg) or vehicle control for 7 consecutive days. Shown are relative mean fluorescence intensity (MFI) of FLICA-YVAD staining in $EpCAM^{+}CD45^{-}$ cells from ileal crypts.

(I-K) Ileal microbiome alteration in intestinal epithelium-specific AIM2 knockout mice. Mice were treated as in (H) and ileal microbiome was collected for 16S rRNA gene sequencing or qPCR analysis. **(I)** Abundance alteration of top-10 abundant bacteria at the genus level. **(J)** Relative proportion of ileal microbiota at the genus level. **(K)** Relative abundance of ileal *SFB* and *Lactobacillus* species measured by qPCR.

(L) Representative images of immunohistochemistry staining of TUNEL. Mice were treated with CPT-Low (20 mg/kg), CPT-Toxic (90 mg/kg) or vehicle control for 7 consecutive days (n = 5 per group) and ileum tissues were collected for analysis.

Data were represented as mean ± SEM. p values were evaluated by two-tailed Student's *t*-test. n.s., not significant; $^{**}p < 0.01$; $^{***}p < 0.001$.

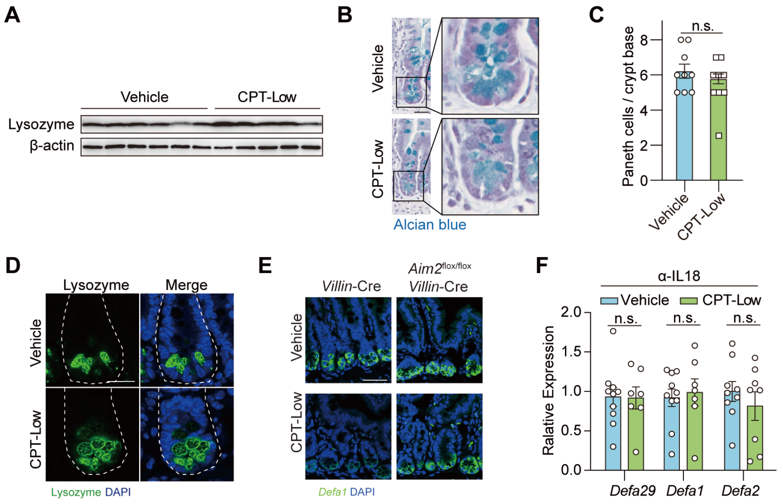

Figure S4. Ileal epithelial AIM2 activation diminishes the antibacterial function of Paneth cells.

(A-D) Paneth cell function analysis. C57BL/6 mice were treated with CPT-Low (20 mg/kg) or vehicle control for 7 consecutive days and ileum tissues were collected. **(A)** Representative images of immunoblot analysis of lysozyme (n = 5 or 6 per group). **(B-C)** Representative images of Alcian blue staining and quantification of Alcian blue positive cells (n = 9 per group). Alcian blue positive cells at the base of ileal crypt were counted and normalized by the crypt counts. Scale bar, 25 μm. **(D)** Representative images of immunohistofluorescent staining of lysozyme at the base of ileal crypts (n = 5 per group). Scale bar, 20 μm.

(E) Paneth cell function in intestinal epithelium-specific AIM2 knockout mice. *Aim2$^{flox/flox}$;Villin-Cre* or littermate control mice (*Villin-Cre*) were treated with low-dose CPT-11 (20 mg/kg) or vehicle control for 7 consecutive days. Shown are representative images of Defa1 mRNA in ileum tissues analyzed by RNA FISH. Scale bar, 50 μm.

(F) Relative expression level of *Defa29, Defa1* and *Defa2*. C57BL/6 mice received anti-IL18 antibody (10 mg/kg) were treated with CPT-Low (20 mg/kg) or vehicle control for 7 consecutive days and ileal crypts were collected for RT-qPCR analysis.

Data were represented as mean ± SEM. p values were calculated by two-tailed Student's *t*-test. n.s., not significant.

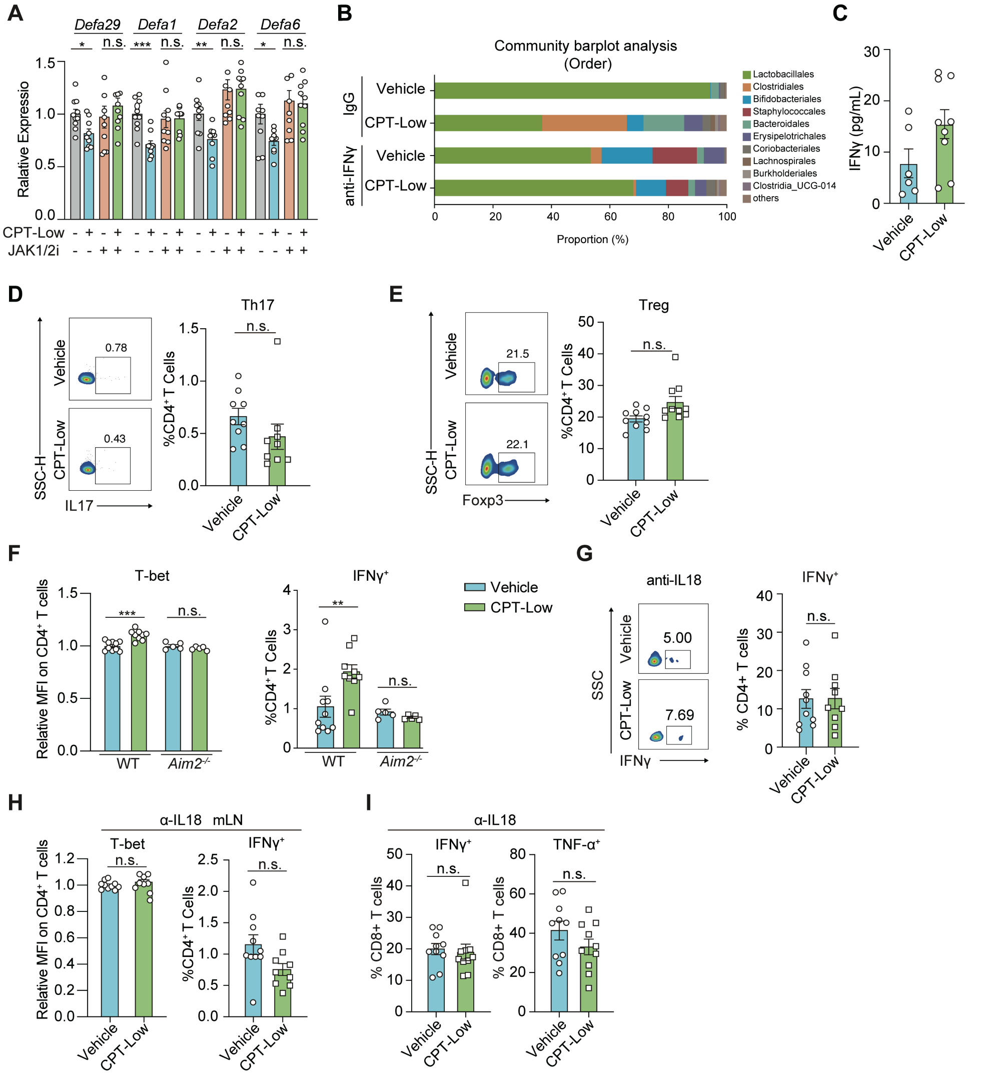

Figure S5. AIM2-dependent IL-18 secretion triggers ileal Th1-Paneth cell interplay to facilitate SFB overgrowth and *Lactobacillus* depletion.

(A) Relative mRNA expression of α-defensins in the ileum. C57BL/6 mice were treated with JAK1/2 inhibitor INCB018424 (100 mg/kg) alone or in combination with CPT-Low (20 mg/kg) for 7 consecutive days (n = 9 or 10 per group) and ileum tissues were collected for qPCR analysis.

(B) Representative bar graph depicting the relative proportion of ileal microbiota at the order level. Mice received anti-IFN-γ antibody (10 mg/kg) were treated with CPT-Low (20 mg/kg) or vehicle control for 7 consecutive days (n = 3 per group), and ileum tissues were collected for 16S rRNA sequencing.

(C) ELISA analysis of IFN-γ concentration in the ileum lysate. C57BL/6 mice were treated with CPT-Low (20 mg/kg) or vehicle control for 7 consecutive days (n = 6 or 9 per group) and ileum tissues were collected for analysis.

(D, E) Th17 and Treg cell ratio in lamina propria-resident CD4^{+} T cells. C57BL/6 mice were treated as in (B) (n = 9 or 10 per group) and ileal lamina propria lymphocytes were isolated for flow cytometry analysis. **(D)** Representative flow cytometry plot and quantification of IL17^{+} cell ratio in

CD4$^+$ T cells. **(E)** Representative flow cytometry plot and quantification of Foxp3$^+$ cell ratio in CD4$^+$ T cells.

(F) Quantification for mean fluorescence intensity (MFI) of T-bet or and IFN-γ$^+$ cell ratio in CD4$^+$ T cells in the mesenteric lymph nodes (mLN). *Aim2*$^{-/-}$ and littermate wildtype (WT) mice were treated as in (B) (n = 9 or 10 per group for WT mice; n = 5 per group for *Aim2*$^{-/-}$ mice).

(G-I) MC38 tumor-bearing mice received anti-IL18 antibody (10 mg/kg) were treated with as in (B) (n = 9 or 10 per group). **(G)** Representative flow cytometry plot and quantification for IFN-γ$^+$ cell ratio in CD4$^+$ T cells in the ileal lamina propria. **(H)** Quantification for mean fluorescence intensity (MFI) of T-bet in CD4$^+$ T cells or IFN-γ$^+$ cell ratio in CD4$^+$ T cells in the mLN. **(I)** IFN-γ$^+$ or TNF-α$^+$ cell ratio in CD8$^+$ T cells in the tumor infiltrated lymphocytes.

Data were represented as mean ± SEM. p values were calculated by two-tailed Student's *t*-test. n.s., not significant; $^{**}p < 0.01$; $^{***}p < 0.001$.

Table S1. Key resources table

**Antibodies**		
β-Actin (8H10D10) Mouse mAb	Cell Signaling Technology	#3700s
GAPDH monoclonal antibody	Proteintech	#60004-1-lg
Cleaved Caspase-1 (Asp296) (E2G2I) Rabbit mAb	Cell Signaling Technology	#89332
Lysozyme antibody (ST50-02)	Novus	NBP2-67507
Anti-dsDNA Antibody	Abcam	#27156
ASC/TMS1 (D2W8U) Rabbit mAb (Mouse Specific)	Cell Signaling Technology	#67824
anti-phospho-Histone H2A.X (Ser139)	Cell Signaling Technology	#9718
anti-Ki67 antibody	Abcam	#ab16667
Anti-ZO1 antibody	Invitrogen	#61-7300
Goat anti-Rabbit IgG (H+L) Cross-Adsorbed Secondary Antibody, Alexa Fluor 488	Invitrogen	A-11008
Goat anti-Mouse IgG (H+L) Cross-Adsorbed Secondary Antibody, Alexa Fluor 488	Invitrogen	A-32723
Goat Anti-Rabbit IgG H&L (Alexa Fluor® 594)	Abcam	Ab150080
FITC-conjugated anti-CD45	BioLegend	#103108
APC/Cyanine7-conjugated anti-CD3	BioLegend	#100222
PerCP/Cyaneine5.5-conjugated anti-CD4	BioLegend	#100434
BV421-conjugated anti-CD4	BioLegend	#100438
PerCP/Cyaneine5.5-conjugated anti-CD8	BioLegend	#45-0081-82
PE-conjugated anti-FOXP3	eBioscience	#12-4771-82
APC-conjugated anti-T-bet	BioLegend	#644813
PE-conjugated anti-IL-17A	eBioscience	#12-7177-81
APC-conjugated anti-IFN gamma	eBioscience	#17-7311-82
PE/Cyanine7-conjugated TNF-alpha	BioLegend	#5066324
InVivoMAb anti-mouse IL-18	BioXcell	#BE0237
InVivoMAb anti-mouse IFN-γ	BioXcell	#BE0055
InVivoMAb anti-mouse PD-1	BioXcell	#BE0146
**Chemicals, peptides, and recombinant proteins**		
Recombinant murine IFN-γ	Peprotech	315-05-20
Mouse Recombinant EGF	Invitrogen	PMG8041
Recombinant Murine Noggin	Peprotech	25038
R-spondin-1	Sino Biological	H-R1-1000
B-27 Supplement Minus Vitamin A	Invitrogen	12587-010
N2 Supplement	Invitrogen	17502-048
N-Acetylcysteine	Sigma	A8199-10G
BD Matrigel ™ Basement Membrane Matrix	BD	356234

Lipofectamine 3000 transfection reagent	Invitrogen	#L30000015
DAPI	Beyotime	#C1002
SN-38	Selleck	S4908
CPT-11	Meilunbio	#MB1126
INCB018424	Meilunbio	#MB5455
neomycin	Meilunbio	#MB1716
vancomycin	Meilunbio	#MB1260
imipenem	Meilunbio	#MB1748
4 kDa FITC-dextran	Sigma	#FD4-1G
EDTA (0.5 M), pH 8.0	Invitrogen	AM9260G
TRIzol® Reagent	Life Technologies	15596018
collagenase IV	Worthington	#LS004188
DNase I	Roche	#10104159001
HEPES	Sigma-Aldrich	83264-100ML-F
Fixable Viability Stain 510	BD	#564406
Fc block	BD	#553141
Cell Stimulation Cocktail	Invitrogen	#00-4975-03
Protease Inhibitor Cocktail	Cell Signaling Technology	#5871s
autoMACS Running Buffer	Meltenyi	130-091-221
20x SSC buffer	Invitrogen	#AM9770
Protease-K	Ambion	#AM2548
Yeast tRNA	Ambion	#AM7119
HiScript II Q Select RT SuperMix for qPCR	Vazyme	#R233
SYBR qPCR Master Mix	Vazyme	#Q711
Advanced DMEM/F-12	Gibco	12634010
Dulbecco's Modified Eagle's Medium	Sigma-Aldrich	#D5030
GlutaMAX™ Supplement	Thermo fisher	35050061
4% Paraformaldehyde	Yeason Biotech	#36314ES76
**Critical commercial assays**		
In Situ Cell Death Detection Kit	Roche	#11684817910
Mouse IL-18 ELISA Kit	Abcam	ab216165
Mouse IL-1β beta ELISA Kit	absin	abs520001-96T
Mouse IFN-γ ELISA Kit	BioLegend	#430807
RNA FISH Kit	GenePharma	
FLICA 660 Caspase-1 Assay Kit	ImmunoChemistry Technologies	#9122
**Experimental models: cell lines**		
MC38	Prof. Yong Cang	
HEK 293T	American Type Culture Collection	
**Experimental models: organisms/strains**		
Aim2$^{-/-}$ mice	Prof Bing Sun	

Lgr5-EGFP-IRES-creERT2 mice	Jackson Laboratory	
C57BL/6 mice	Beijing Vital River Laboratory Animal Technology	
BALB/c nude mice	Beijing Vital River Laboratory Animal Technology	
Aim2$^{flox/flox}$ mice	Gempharmatech	
Villin-Cre mice	Gempharmatech	
**Oligonucleotides**		
FAM-5'GTCCTCTTCTCCTGGCTGCTCCTCA-3’	GenePharma	
FAM-5’ ATACGGCCTGGTCCTCTTCTCCTGG-3'	GenePharma	
5'-FAM-CAATAGCATACCAGATCTCTCAACG-3'	GenePharma	
EUB338: FAM-5' GCTGCCTCCCGTAGGAGT -3	Sangon	
SFB: Cy3-5’ GGGTACTTATTGCGTTTGCGACGGCAC -3’	Sangon	
**Recombinant DNA**		
pCMV6-Entry Mammalian Expression Vector	OriGene	PS1000001
pCMV-*ASC* CDS Vector	This paper	
pCMV-*Aim2* CDS Vector	This paper	
**Software and algorithms**		
NDP.View 2 software	Hamamatsu	
Prism software	Graphpad	
R 4.0.2	R Development Core Team	https://www.r-project.org
ImageJ software (Fiji)	(Schindelin et al., 2012)	https://fiji.sc
FlowJo	BD	
**Others**		
Leica TCS SPS CFSMP	Leica	
ImageQuantTM LAS 4000	GE Healthcare	
Olympus IX73 microscope	Olympus	
BD LSRFortessa X-20	BD	

Table S2. The primer sequences.

**Primers used in RT-qPCR**		
Gene	Forward	Reverse
Mouse *Hprt*	TGCTCGAGATGTCATGAAGGAG	CAGAGGGCCACAATGTGATG
Mouse *Defa1*	TCAAGAGGCTGCAAAGGAAGAGAAC	TGGTCTCCATGTTCAGCGACAGC
Mouse *Defa2*	CCAGGCTGATCCTATCCAAA	GTCCCATTCATGCGTTCTCT
Mouse *Defa6*	CCTTCCAGGTCCAGGCTGAT	TGAGAAGTGGTCATCAGGCAC
Mouse *Defa29*	CACCACCCAAGCTCCAAATACACAG	ATCGTGAGGACCAAAAGCAAATGG
Mouse *Ifng*	GCTTTGCAGCTCTTCCTCAT	CCAGTTCCTCCAGATATCCAAG
**Primers used in qPCR for ileal SFB and *Lactobacillus* analysis**		
SFB	AGGAGGAGTCTGCGGCACATTAGC	TCCCCACTGCTGCCTCCCGTAG
L. Johnsonii	CACTAGACGCATGTCTAGAG	AGTCTCTCAACTCGGCTATG
L. Murinus	TCGAACGAAACTTCTTTATCACC	CGTTCGCCACTCAACTCTTT
L. Reuteri	ACCGAGAACACCGCGTTATTT	ACCTAAACAATCAAAGATTGTCT
Mouse Gapdh	CATCACTGCCACCCAGAAGACTG	ATGCCAGTGAGCTTCCCGTTCAG
